# Identification of the vernalization gene *VRN-B1* responsible for heading date variation by QTL mapping using a RIL population in wheat

**DOI:** 10.1186/s12870-020-02539-5

**Published:** 2020-07-13

**Authors:** Yuting Li, Hongchun Xiong, Huijun Guo, Chunyun Zhou, Yongdun Xie, Linshu Zhao, Jiayu Gu, Shirong Zhao, Yuping Ding, Luxiang Liu

**Affiliations:** Institute of Crop Sciences, Chinese Academy of Agricultural Sciences/National Engineering Laboratory for Crop Molecular Breeding, National Center of Space Mutagenesis for Crop Improvement, Beijing, China

**Keywords:** Heading time, Wheat, BSR-Seq, *Vrn-B1*, Genetic mapping, Metabolic pathways

## Abstract

**Background:**

Heading time is one of the most important agronomic traits in wheat, as it largely affects both adaptation to different agro-ecological conditions and yield potential. Identification of genes underlying the regulation of wheat heading and the development of diagnostic markers could facilitate our understanding of genetic control of this process.

**Results:**

In this study, we developed 400 recombinant inbred lines (RILs) by crossing a γ-ray-induced early heading mutant (*eh1*) with the late heading cultivar, Lunxuan987. Bulked Segregant Analysis (BSA) of both RNA and DNA pools consisting of various RILs detected a quantitative trait loci (QTL) for heading date located on chromosomes 5B, and further genetic linkage analysis limited the QTL to a 3.31 cM region. We then identified a large deletion in the first intron of the vernalization gene *VRN-B1* in *eh1,* and showed it was associated with the heading phenotype in the RIL population. However, it is not the mutation loci that resulted in early heading phonotype in the mutant compared to that of wildtype. RNA-seq analysis suggested that *Vrn-B3* and several newly discovered genes, including beta-amylase 1 (*BMY1*) and anther-specific protein (*RTS*), were highly expressed in both the mutant and early heading pool with the dominant *Vrn-B1* genotype compared to that of Lunxuan987 and late heading pool. Enrichment analysis of differentially expressed genes (DEGs) identified several key pathways previously reported to be associated with flowering, including fatty acid elongation, starch and sucrose metabolism, and flavonoid biosynthesis.

**Conclusion:**

The development of new markers for *Vrn-B1* in this study supplies an alternative solution for marker-assisted breeding to optimize heading time in wheat and the DEGs analysis provides basic information for *VRN-B1* regulation study.

## Background

Wheat is one of the most widely cultivated food crops worldwide, and successful adaptation of this important grain crop in different agro-ecological conditions is mainly determined by flowering and maturity time. Heading date is tightly connected with crop maturity and production. Identification and isolation of genes related to heading date variation will provide better understanding of the genetic pathways that control flowering time in plants and offer effective genetic resources for engineering high-yield varieties that can adapt to various conditions [1].

Plants have evolved multiple genetic pathways to regulate the flowering pathway, including vernalization requirements and photoperiod sensitivity, integrating both external and internal signals to adapt to changes in climatic conditions. Vernalization is the requirement of exposure to prolonged periods of low temperature that provides competence to flower in many plant species adapted to temperate climates [2]. In wheat, the vernalization-induced flowering pathway is mainly regulated by the *VERNALIZATION* gene series: *VRN1* [3], *VRN2* [4], *VRN3* [5] and *VRN-D4* [6]. Identification and characterization of these genes furthered our understanding of vernalization-mediated flowering regulatory networks in cereals. The MADS-box transcription factor *VRN1* gene, was first identified in *Triticum monococcum* and functions in acceleration of flowering after vernalization [3]. *VRN2* encodes a zinc-finger-CCT domain transcription factor and represses the flowering in cereals [4, 7, 8]. The CCT domain in *VRN2* is a key element for vernalization in both wheat and barley, and mutation of the CCT domain eliminates a vernalization requirement [9, 10]. *VRN3* is homologous to the *Arabidopsis* photoperiod gene *FLOWERING LOCUS T (FT)* and its gene product functions as a mobile signaling protein, moving from leaves to the shoot apical meristem to accelerate flowering [5]. *VRN-D4* originated from an insertion of a ~ 290 kb region from chromosome arm 5AL into the proximal region and contains a copy of *VRN-A1* [6].

*VRN1* has been well reported in wheat and is orthologous to the *Arabidopsis* floral meristem-identity genes *CAULIFLOWER (CAL), APETALA1 (AP1)*, and *FURITFULL (FUL)* [3, 11–14]. In hexaploid wheat, a homoeologous copy of the *VRN1* gene is present in the proximal regions of chromosomes 5A, 5B, and 5D, and have been designated *VRN-A1*, *VRN-B1* and *VRN-D1*, respectively. Allelic variation at the *VRN1* locus is one of the main resources of genetic variation in vernalization requirements in wheat. A single dominant allele of one of the three homeoloci is sufficient to confer a spring growth habit [15–17]. A previous study showed that the dominant *Vrn1* with spring growth habit contained large deletions in the first intron, and a 2.8-kb conserved segment within the deletion region of recessive *vrn1* was important for vernalization requirement in wheat [16]. Additionally, the investigation of polymorphisms in the RNA immune precipitation fragment 3 (RIP3) of *VRN-A1* first intron suggested that single nucleotide polymorphisms in RIP3 affected *VRN-A1* transcript level, and are associated with differences in heading time of winter wheat cultivars from various geographic regions [17]. The differences between dominant and recessive alleles associated with the *VRN-B1* locus lie near the first intron and mainly include a large deletion, SNPs, small deletions, and large insertion in the 5′-UTR region [16, 18–22].

The *VRN1*–*VRN2*–*VRN3* regulatory feedback network integrates vernalization and photoperiod signals to precisely mediate the flowering time of wheat. In barley, high levels of *VRN2* repress the expression of *VRN3*, inhibiting flowering. In wheat, the up-regulation of *VRN1* transcriptional levels results in decreased expression levels of flowering repressor *VRN2* after vernalization, which accelerates the induction of *VRN3* expression levels, and thus induces flowering in the spring [23, 24]. Epistatic analysis indicated that *VRN2* interacts with *VRN1* and shares a genetic pathway [25]. *VRN3* up-regulates the expression of *VRN1* through interactions with FDL2, a basic leucine-zipper (bZIP) transcription factor [26]. FDL2 is orthologous to the bZIP transcription factor FD in *Arabidopsis* that binds ACGT elements in the promoter of *VRN1* [26, 27]. The expression of *VRN3* can also be affected by competition with *CO* and *VRN2* to interact with the nuclear factor-Y (NF-Y) complex, which plays an important role in the integration of vernalization and photoperiod seasonal signals [28].

Many QTL have been identified for controlling heading time in wheat. Until now, more than 240 QTL related to heading time have been detected in wheat [29, 30], and are important for marker-assisted selection in wheat breeding. However, most of genes underlying these loci remain unknown. In this study, we identified a QTL for heading time located on chromosome 5B by Bulked Segregant Analysis (BSA) and genetic linkage mapping, and showed that the mapped region contained the *VRN-B1* gene that is involved in heading time variation in the RIL population but it is not the reason for early heading phenotype in the mutant (*eh1*) compared to that of wildtype (WT, Zhongyuan9). In order to identify genes regulated by *VRN-B1,* we identified genes that were differentially expressed between the early and late heading bulks, and investigated their associated pathways. This study provides important clues for marker assisted breeding for heading time in wheat and expands our knowledge on genetic regulation of the *VRN-B1* gene.

## Results

### Construction of the RIL population and heading time variation

**Fig. 1:**
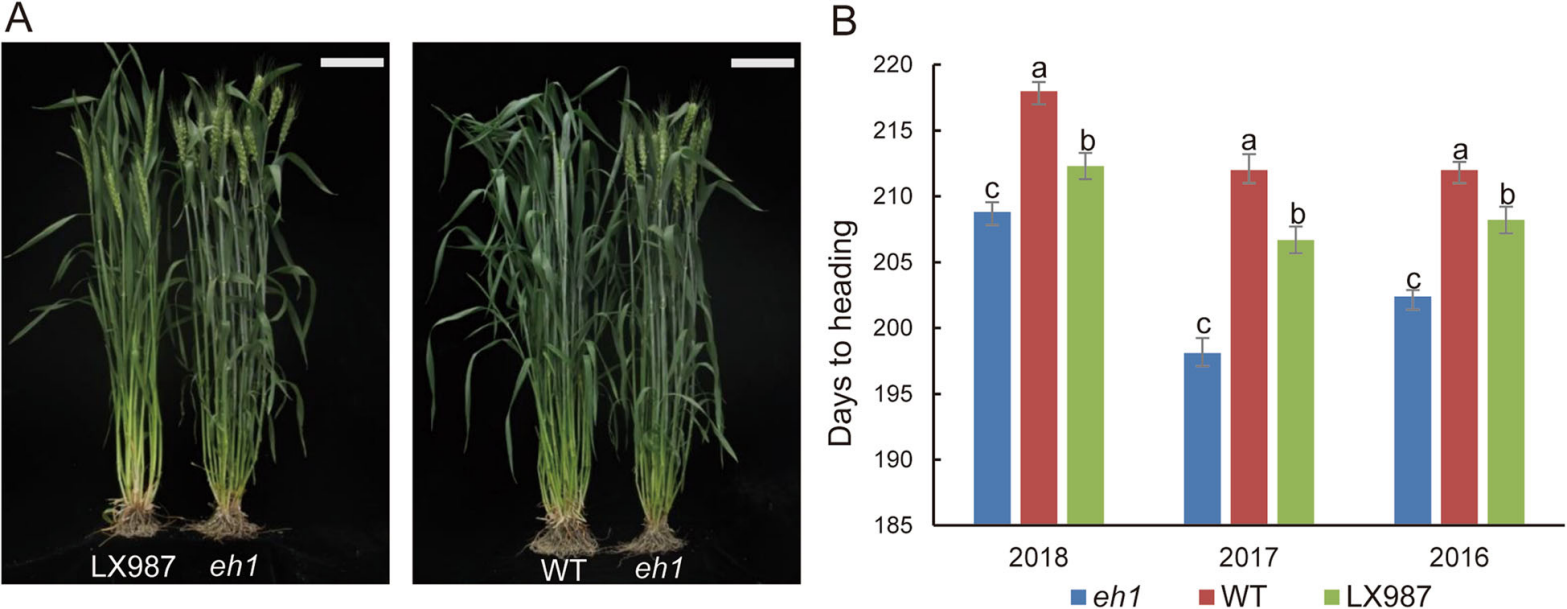
Phenotypic analysis of wheat lines used in this study. **a** Phenotypic comparison between the late heading parental line (LX987) and the early heading mutant *eh1* (left). WT and *eh1* at the heading stage (right). Plants were grown under field conditions and the photos were taken on April 27th, 2017. Bars = 10 cm. **b** Days to heading of the *eh1*, WT, and LX987 with about 10 rows of plants were investigated in 2016, 2017 and 2018. Values are means ± SD and different letters indicate the significant difference between the comparison groups at *P* < 0.05

The early heading mutant *eh1* was obtained by mutagenesis of seeds of Zhongyuan9 wheat lines with 284 Gy γ-ray treatment. To map the QTL responsible for heading date (HD) variation, we developed 400 recombinant inbred lines (RILs) by crossing *eh1* with Lunxuan987 (LX987), an elite cultivar with a minor defect of late heading for agricultural practice. The phenotype comparison between LX987 and *eh1*, WT and *eh1* was shown in Fig. 1a. The HD of *eh1* was 10–14 days earlier than WT over 3 years under field conditions (Fig. 1b, Table S1). The HD of LX987 is 4–8 days later than that of *eh1* with significantly statistical difference (Fig. 1b, Table S1). Measurement of HD in RILs over a period of 3 years suggested that the maximum HD is 215 days and the minimum HD is 197 days (Tables S1, S2). Additionally, HD in the RIL population showed a continuous distribution (Fig. S1), indicating the flowering time in the RIL is a quantitative trait.

### Mapping of an early heading gene using a BSR-seq approach

Bulked Segregant RNA-Seq (BSR-Seq) is an efficient approach for gene mapping [31], especially in wheat plants that have large genomes. We performed BSR-Seq to map the QTL controlling HD in the RIL population. Young spikes of 21 early heading and 22 late heading lines, based on HD data collected over a 3 year period, were sampled when the early heading lines started to heading. The RNA of individual samples was isolated and then early or late heading RNA bulks were constructed. Meanwhile, young spikes of six individuals for two parent lines were also sampled for RNA-Seq analysis. In total, 357,941 SNPs were obtained from two parents and two bulks by RNA-Seq. A total of 160 SNPs with multiple alleles and 180,469 loci with reads < 4 were filtered out. Sixty-nine thousand two hundred twenty-two SNPs with the same genotype and 61,288 SNPs from bulks that differed from those in corresponding parents were also discarded. Finally, 46,802 reliable, high-quality SNPs distributed across all chromosomes were identified and used to perform further analysis.

**Fig. 2:**
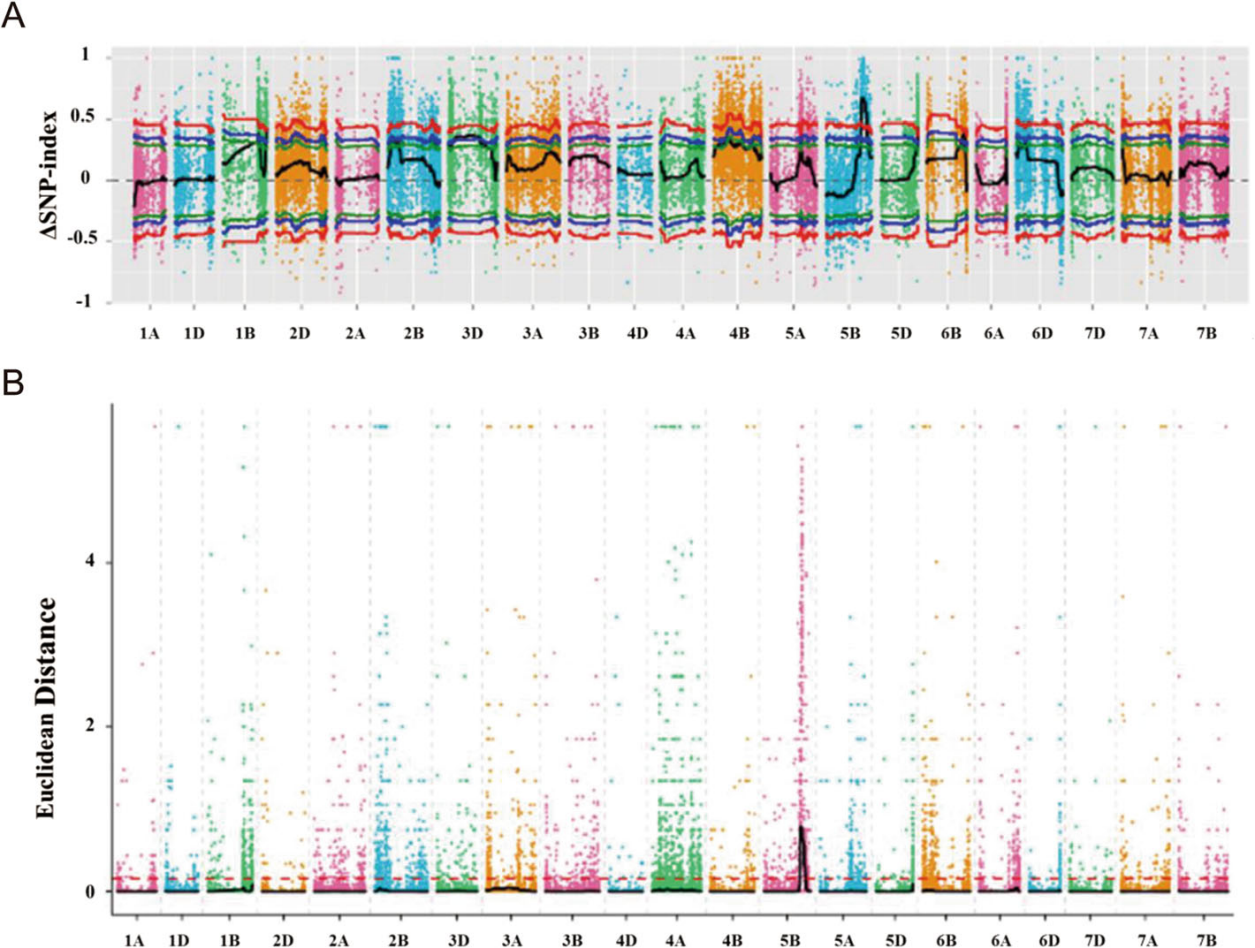
Association mapping by BSR-Seq from two extremely bulks and two parent lines. **a** The distribution of ΔSNP-index values across all chromosomes. Black lines represent the fitted ΔSNP-index values. Red, blue and green lines represent the significant levels at 0.99, 0.95 and 0.90 confidence, respectively. The x-axis indicates the name of all chromosomes and the y-axis indicates the SNP-index value. **b** The distribution of ED-association values across all chromosomes. The color points represent the ED value of each SNP locus. The black line and red dotted line represent the fitted ED value and the significant correlation threshold, respectively. The higher the ED value, the better the association effect

The Δ (SNP-index) was calculated based on the reads and the genotypes of bulks and parents. The results showed that the region of 546.9 Mb - 636.6 Mb on chromosome 5B with a SNP-index peak was the major QTL associated with HD in the RIL population (Fig. 2a). Calculation of the Euclidean Distance (ED) value was also used to identify loci controlling HD, and suggested that a total of 1113 genes were present in an 89.57 Mb region on chromosome 5B according to the gene information of Chinese Spring reference sequence (http://www.wheatgenome.org/) (Fig. 2b). Both SNP-index and ED analyses detected a common interval on chromosome 5B, suggesting that an 89.57 Mb region of chromosome 5B harbors the QTL responsible for heading variation in the RILs.

### The same region on chromosome 5B associated with HD was detected by BSA consisting of various RILs

Based on 2 years phenotype data, three extremely early heading bulks consisting of 18–22 early heading lines for each bulk, and three extremely late heading bulks consisting of 16 late heading lines, respectively, were used for BSA analysis. A total of 6 bulks and 2 parents were genotyped by using 660 K SNP array. The SNPs showing different genotypes in a pair of early and late heading bulks, and the same genotypes between bulk and parent with the same phenotype, were selected and the SNP numbers among 21 chromosomes were counted. The results showed that a large number of SNP were detected on chromosomes 2A and 5B between the early and late heading bulk 1 (Fig. S2A), on chromosomes 5B, 2B and 3A between the early and late heading bulk 2 (Fig. S2B), on chromosomes 3B, 5B, 2B and 6A between the early and late heading bulk 3 (Fig. S2C), suggesting QTLs controlling HD exist on these chromosomes. The SNP frequencies distributed on chromosome 5B were analyzed. High peak regions from 540 to 600 Mb on chromosome 5B overlapped in the three pairs of bulks were observed (Fig. S2D), which is consistent with the BSR-Seq analysis.

### Confirmation of the region by genetic mapping

**Fig. 3:**
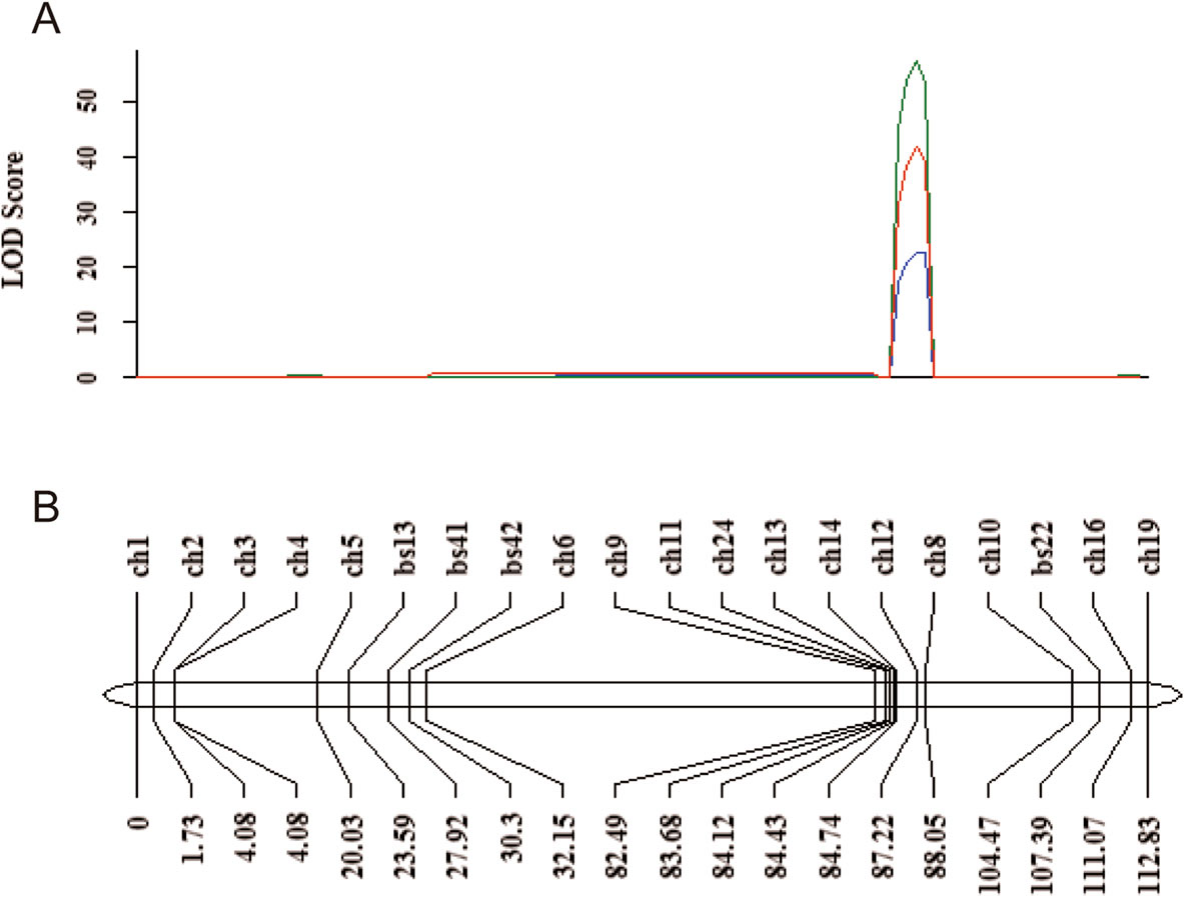
Linkage map and QTL mapping of the early heading variation in RILs on chromosome 5B. **a** QTL curve of logarithm of odds (LOD) scores calculated for three individual years. The LOD value, located on 3.31 cM of the genetic linkage map between markers *ch14* and *ch8*, peaked at 41.7, 57.4, and 22.8 in 2016, 2017 and 2018, respectively. Each QTL explained a phenotypic variation of 36.9, 46.6, 22.6% in 2016, 2017 and 2018, respectively. **b** Linkage map of 20 KASP markers on chromosome 5B. The entire map distance was 112.8 cM and the average genetic distance was 5.4 cM

To validate the BSA-based mapping region, 87 RNA-Seq-derived SNPs on chromosome 5B were selected for development of KASP markers. Specific SNPs on the three wheat genomes were selected using PolyMarker [32], confirmed on parents and population lines, then 20 specific KASP markers were finally developed (Table S3). The genotypes of 400 lines in the RIL population were identified. We generated a linkage map spanning 112.83 cM on chromosome 5B, with an average genetic distance of 5.6 cM, using QTL IciMapping (Fig. 3). Based on the combined genetic and phenotypic data generated over 3 years, a stable QTL with a LOD score of more than 22.8 was detected in the region between the two markers, *ch14* and *ch8*, with a genetic distance of 3.31 cM, corresponding to the physical position of 560.4–580.1 Mb (Fig. 3).

### The *Vrn-B1* is responsible for HD variation in the RILs

**Fig. 4:**
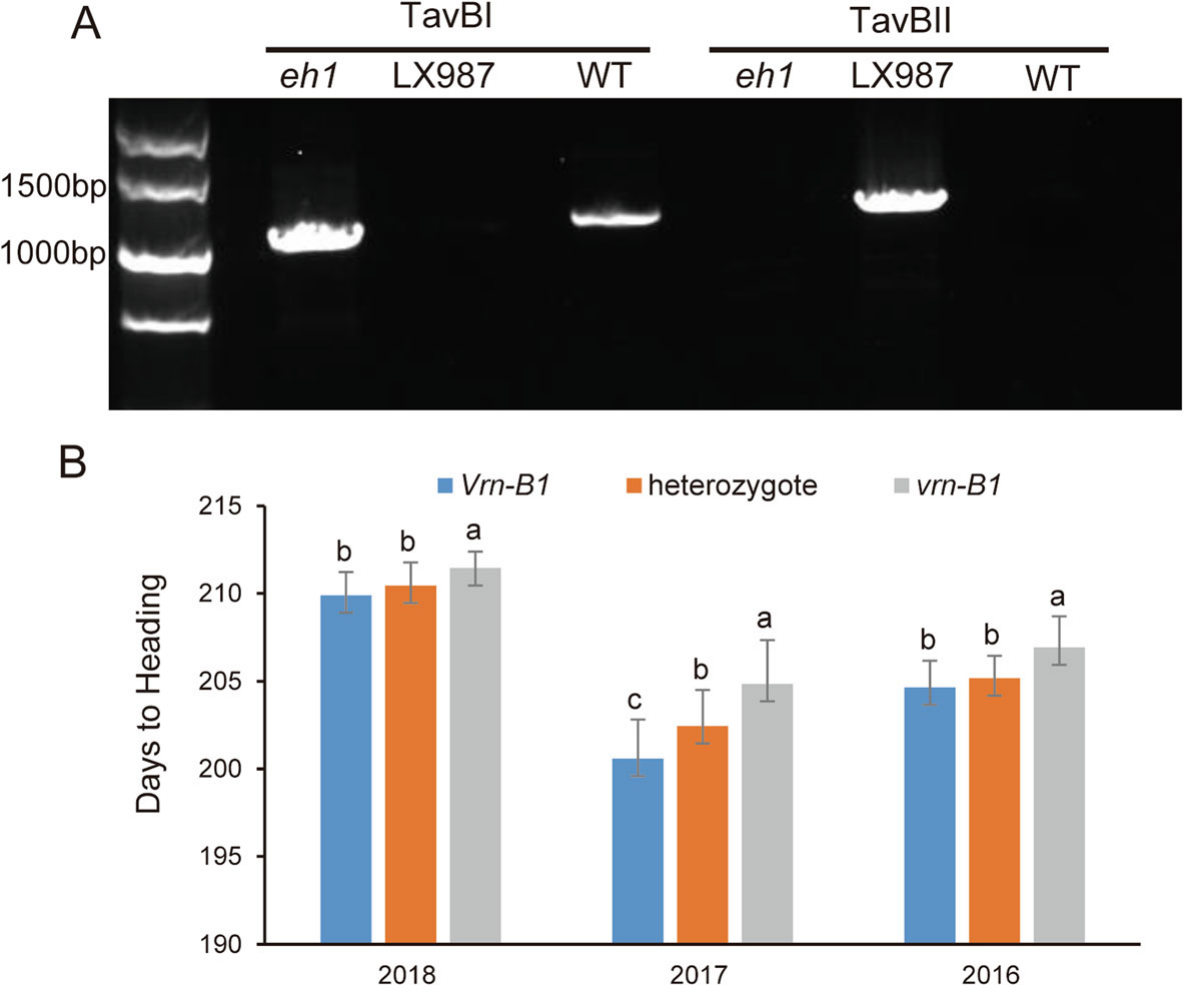
Development of gene-based markers for *VRN-B1* and heading time variation of RILs with different *VRN-B1* alleles. **a** Identification of a deletion in *VRN-B1* by two complementary primers. Products were detected in *eh1* and WT by TavBI but not by TavBII, indicating that this region was absent in both the *eh1* and WT. Full-length gels are presented in Supplementary Figure 9. **b** Days to heading of RILs with different *VRN-B1* alleles in 2016, 2017 and 2018. Values are means ± SD. Different letters indicate statistically significant difference among the comparison groups at *P*< 0.05

The vernalization gene *VRN-B1* located on chromosome 5B is a major locus associated with wheat heading and flowering [3]. In natural variants, the dominant *Vrn-B1* allele with spring growth habit differs from the recessive allele *vrn-B1* with winter growth habit due to a large deletion in the first intron of *Vrn-B1* [16]. We found that the mapped region on chromosome 5B contained the *VRN-B1* gene. To test whether the major QTL on chromosome 5B controlling HD is associated with the *Vrn-B1* gene, we used the markers developed by previous study [16] but failed to get the PCR product in WT and *eh1*. Therefore, specific markers for *Vrn-B1* were developed (Fig. S3A, Table S3). For the *VRN-B1* gene markers, two pairs of genome-specific primers were designed to determine whether a large deletion in the first intron of *Vrn-B1* was absent or present (Fig. S3A)*.* Amplification products were detected both in *eh1* and WT, but were not produced using DNA isolated from LX987 using the TavBI primer pair. However, a PCR product was amplified from LX987 DNA with the TavBII primer pair (Fig. 4a), indicating that the large deletion exists in *eh1* and WT but does not in LX987. The genotypes of *VRN-B1* in the *eh1*, WT and LX987 suggested that the genotypic differences observed in *VRN-B1* in the RIL population resulted from genetic background diversity between WT and LX987, but not from the γ-ray irradiation. We then identified the genotypes of *VRN-B1* in the RIL population, and showed that 199 RILs with the *Vrn-B1* allele displayed significantly (*P* < 0.001) earlier heading dates than 185 RILs that possessed the *vrn-B1* allele. The *Vrn-B1* lines headed approximately 2.7 days earlier than *vrn-B1* lines on average (Fig. 4b). In addition, the heading time of the heterozygotes was 206 days, and the degree of dominance is 0.28, indicating that *Vrn-B1* has a small dominant effect. Further sequencing the *VRN-B1* in WT, *eh1* and LX987 found that the A changed to G in the 3′ end and C changed to A in the middle of primer Intrl/B/F [16] in WT and *eh1* (Fig. S3A, B). In addition to the large deletion in the first intron of WT and *eh1*, a 37 bp deletion located downstream of the large deletion was only detected in *eh1* (Fig. S3A). We then developed specific markers for the 37 bp deletion (Fig. S3C) and identified the genotypes in the RIL population, and found that the 37 bp deletion co-segregated with the large deletion. Further analysis using an F_2_ population containing 1060 individuals derived from cross of *eh1* and WT indicates that the 37 bp deletion is not associated with HD variation (Fig. S4, *P* = 0.757).

To identify if natural variation of *VRN-A1* existed in *eh1* and LX987 affecting heading time, we used previously developed markers to distinguish dominant *Vrn-A1* and recessive *vrn-A1* [16]. The results showed that both *eh1* and LX987 harbored the recessive *vrn-A1* allele (Fig. S5A). *Ppd-A1* is an important regulator of heading time that located on chromosome 2A. To determine the sequence variations of *Ppd-A1* gene between *eh1* and LX987, we sequenced the *Ppd-A1* gene as well as its up- and down-stream region. Sequence comparison showed no variations in the coding region of *Ppd-A1* gene among *eh1*, WT and LX987 (Fig. S5C). A 131 bp deletion on the 3′ region of *Ppd-A1* was detected in *eh1* and this variation also existed in Chinese Spring (Fig. S5B, C). We also compared the heading time of lines carrying different variations at this locus in the RIL population and found that the 131 bp deletion did not affect heading time (Fig. S5D).

### The difference of heading time between *eh1*, WT, and LX987 under vernalization or non-vernalization conditions

**Fig. 5:**
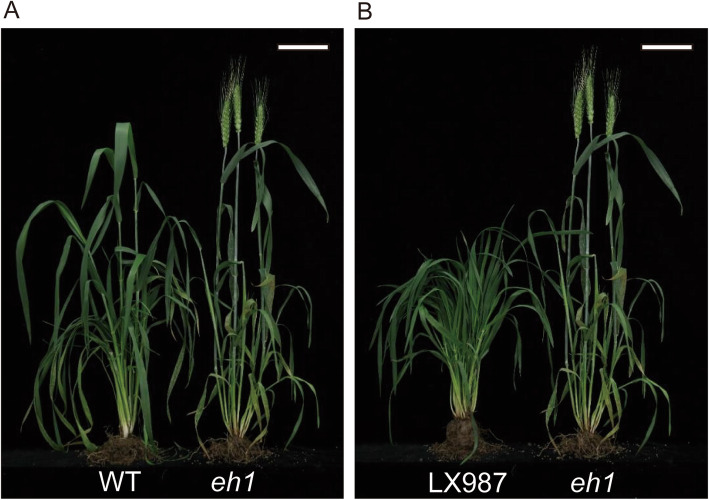
Phenotypic analysis of wheat lines cultivated under non-vernalization condition. **a** WT and *eh1*, **b** LX987 and *eh1* planted without vernalization process. LX987 failed to flower in the full life cycle. Bars = 10 cm

To investigate whether the mutated early heading gene is related to vernalization, the heading time of *eh1*, WT and LX987 lines was compared starting with seeds that were or were not subjected to vernalization. When seeds were sown in autumn (vernalized during winter), during 3 years investigation the *eh1* headed 10–14 days and 4–8 days earlier than WT and LX987 with significantly statistical difference, respectively [22]. When seeds were sown in spring without vernalization, LX987 failed to flower and *eh1* headed approximately 13 days earlier than WT (Table 1, Fig. 5). A similar variation in heading date between *eh1* and WT, with or without vernalization, suggested that the early heading phenotype observed for *eh1* did not depend on vernalization.

**Table 1:** HD of *eh1*, WT and LX987 wheat lines grown under vernalization or non-vernalization conditions

Lines	HD (vernalized)^a^	HD (unvernalized)^b^
*eh1*	203.1 ± 4.55	68 ± 0.82
WT	214 ± 2.82	81.7 ± 0.58
LX987	209.1 ± 2.59	–

^a^ When seeds of *eh1*, WT and LX987 lines were sown in autumn, the wheat plants were vernalized during winter and HD from at least three rows for each lines were investigated. Values are means ± SD. ^b^ Seeds were sown in spring without vernalization and HD from at least three rows for each lines were investigated. Values are means ± SD. LX987 failed to flower under non-vernalization condition

### Identification of differentially expressed genes (DEGs) by RNA-seq

**Fig. 6:**
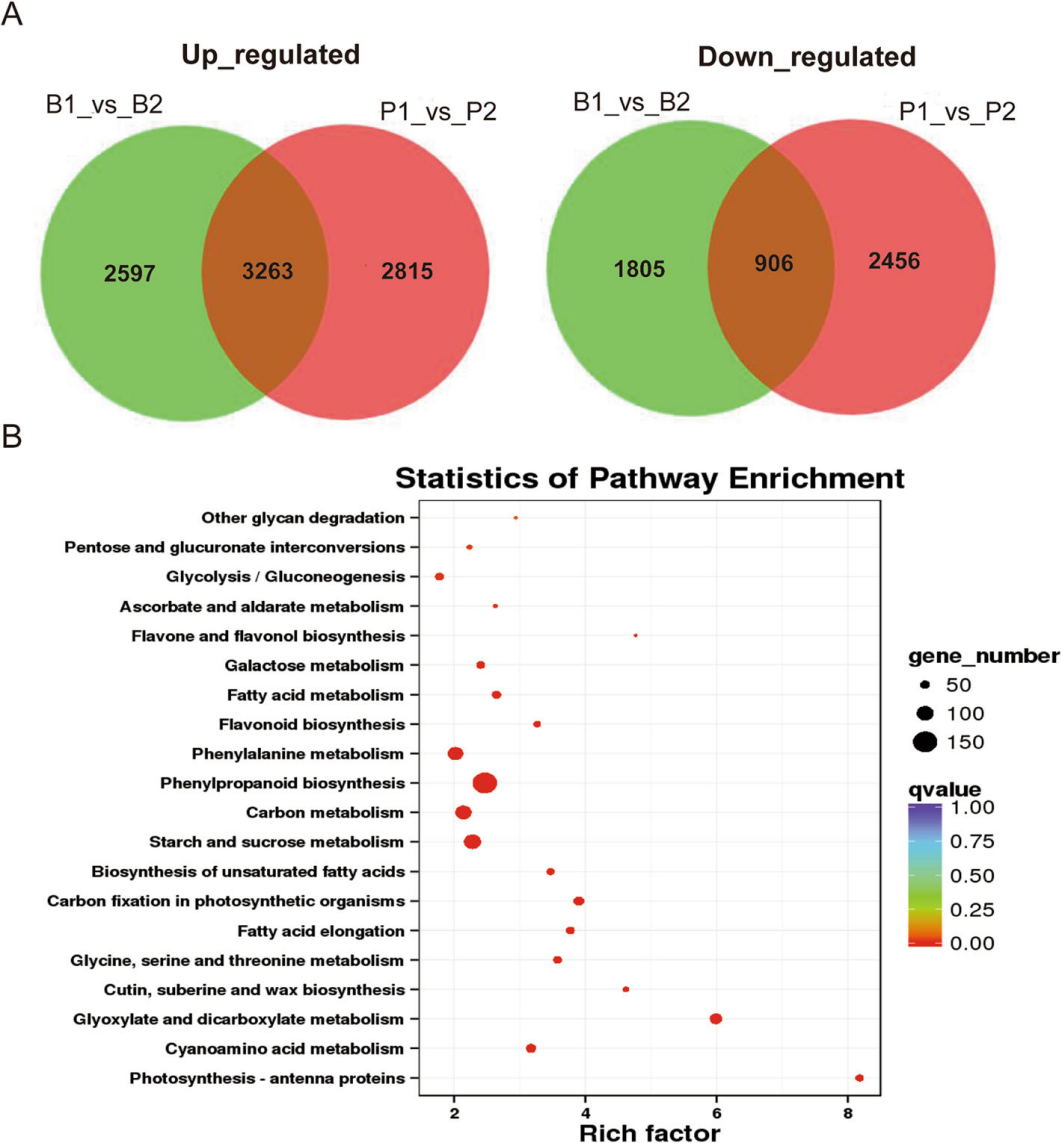
Analysis of DEGs. **a** Venn diagrams illustrating the overlap in DEGs between the two heading bulks and parents. B1 and B2 indicate the early heading bulk and the late heading bulk, respectively, while P1 and P2 represent the early heading mutant and LX987, respectively. The number in each circle represents the total number of DEGs in each comparison group, and the number in the overlapping areas indicates the number of shared DEGs between two comparison groups. **b** KEGG pathways enriched in the DEGs overlapped in the B1_vs_B2 and P1_vs_P2 groups. The number of DEGs in each listed pathway is indicated by size the circle area, the rich factor reflects the degree of enriched DEGs in a given pathway and the circle color represents the range of the corrected *P* values

The genotype of *Vrn-B1* in RILs suggested that all the RILs used for the early heading pool harbored a dominant allele (*Vrn-B1*) and 21 of the 22 RILs used for the late heading pool were recessive (*vrn-B1)* (Table S4). RNA-Seq data, to some extent, can be reliably used to investigate the transcriptomic changes due to the presence of different *VRN-B1* alleles. Based on our criteria (fold change ≥2 and FDR < 0.05), a total of 9440 DEGs were identified between the two parents, in which 6078 were up-regulated and 3362 were down-regulated (Table S5). A total of 8571 DEGs, including 5860 up-regulated and 2711 down-regulated genes, were identified between early and late heading pools (Table S5). Among all the DEGs, 4169 genes overlapped between the comparison groups of two parents and two bulks, including 3263 up-regulated and 906 down-regulated genes (Fig. 6a, Table S5). Among them, 103 up-regulated genes, including beta-amylase 1 (*BMY1,* TraesCS2B01G240100), 3-oxoacyl-[acyl-carrier-protein] synthase I (*KAS12,* TraesCS2B01G156800) and anther-specific protein (*RTS,* TraesCS3D01G436700), with a fold change > 100 and *P* < 0.0001 were identified (Table S5). Additionally, we found the vernalization gene *VRN-B3* (TraesCS7B01G013100) was up-regulated (*P* < 0.01) in both *eh1* and the early heading bulk sample compared to LX987 and late heading bulk with a fold change of 4.63 and 3.36, respectively.

### Expression comparison of selected DEGs and vernalization genes by RT-qPCR

**Fig. 7:**
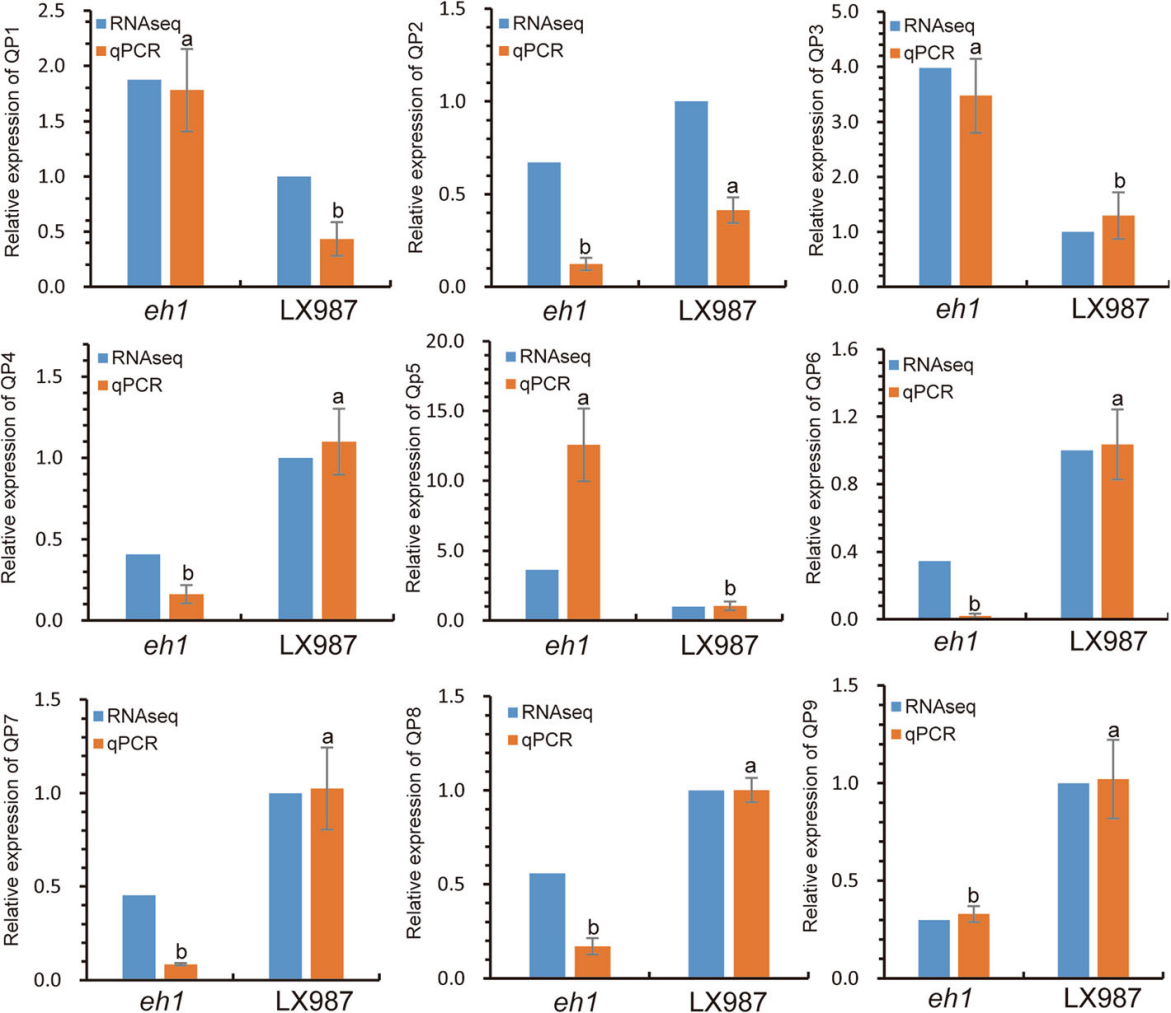
Real-time qPCR verification of selected genes in *eh1* and LX987. The detailed information of genes labeling with QP1 to QP9 is shown in Table S7. The RNA-seq values (blue) represent the ratio of expression fold change in *eh1* relative to LX987. Data generated by qPCR (orange) are shown as means ± SD from three biological replicates and different letters indicate significant differences of gene expression levels between *eh1* and LX987 by qPCR at *P* < 0.05

To verify the reliability of gene expression levels generated by RNA-seq, nine genes including the *Vrn-B3*, were selected for validation using real-time quantitative PCR analysis. As shown in Fig. 7, the relative expression levels of selected genes by RT-qPCR analysis were similar with the expression trends observed in the RNA-seq data, with a high Pearson’s correlation coefficient (*R*^*2*^ = 0.79).

To compare the expression levels of vernalization genes between *eh1* and LX987, RT-qPCR were used to quantify the relative expression of *VRN-A1*, *VRN-B1*, *VRN-D1*, and *VRN2* genes. When *eh1* started to heading, young spikes and leaves of *eh1* and LX987 from 10 replicates were sampled, respectively. The expression levels of *VRN-A1*, *VRN-B1* and *VRN-D1* were significantly higher in the young spikes of *eh1* compared to that of LX987 (Fig. S6). In the leaves, the expression amount of *VRN-B1* and *VRN-D1* were also significantly higher in *eh1* than in LX987 (Fig. S6). In contrast, the expression levels of *VRN2* gene in both young spikes and leaves of *eh1* were significantly lower than that of LX987 (Fig. S6). To analyze the expression levels of these genes in *eh1* and LX987 at the same developmental stage, young spikes and leaves of *eh1* were also sampled 8 days earlier than that of LX987 when they showed similar developmental levels of spikes. A completely opposite results were obtained for the samples from the same developmental stage compared to that sampling at the same day (Fig. S7).

### GO and KEGG pathway enrichment analysis

Gene ontology (GO) enrichment analysis was performed to investigate the functions of overlapped DEGs between two parents and two bulks. In total, 1537 GO categories were enriched and 42 of them were significantly enriched based on the corrected *P*-value < 0.05 (Table S6). The top 20 significantly enriched GO terms suggested that biological processes including photosynthetic electron transport in photosystem I, reductive pentose-phosphate cycle, carbohydrate metabolic process and fatty acid biosynthetic process, were affected by changes in heading time resulting from *VRN-B1* variation (Table S6). KEGG pathway enrichment of the overlapped DEGs indicated that significantly enriched pathways related to photosynthesis, including antenna proteins, fatty acid elongation, carbon fixation in photosynthetic organisms, starch and sucrose metabolism, carbon metabolism, and fatty acid metabolism, are involved in heading process (Fig. 6b, Table S7).

## Discussion

Variability of HD is of great significance for the adaptation of wheat cultivars to local environments, and its optimization assists yield improvement during the wheat breeding process [33]. Induced mutation is an effective approach for creation of new germplasm [34]. The γ-ray induced early heading wheat mutant, *eh1,* used in this study provides novel genetic variation for HD. By using *eh1* and the late heading cultivar LX987 as parents, a RIL population was constructed for BSA and genetic mapping analysis. By combining BSA and genetic mapping approaches, a stable QTL for HD with a LOD score exceeding 22.8 was detected, and flanking markers were located on a 20 Mb interval that was found to include the *VRN-B1* locus [3]. Further analysis using two genome-specific primers suggested that the presence or absence of a large deletion in the first intron of *VRN-B1* was associated with heading time variation in the RIL population. Consistent with these results, a previous study suggested that large deletions within the first intron of *VRN1* resulted in a spring growth habit and affected flowering time [16]. Additionally, a 2.8 kb region in the first intron of *vrn-B1* present in different recessive alleles showed high sequence conservation [16, 35]. It was assumed that this critical region contained a binding site for a putative repressor that is down-regulated by vernalization; thus, the presence of large deletion in the first intron of *Vrn-B1* ablates suppressor function on *VRN-B1* [16, 22, 35]. Due to the different genotypes of *VRN-B1* between the two parents of the RIL population in this study, it is reasonable to identify the major QTL (Figs. 2, 3). By using the two pairs of *Vrn-B1* gene markers, we found that the lines used for construction of the early and late heading bulks for BSR-Seq were almost divided equally between *Vrn-B1* and *vrn-B1*, respectively (Table S4), indicating that the results from BSR-Seq are reliable. The same genotypes of large deletion in the first intron of *VRN-B1* between WT and *eh1* (Fig. 4a), combined similar variations of HD between WT and *eh1* with or without vernalization (Fig. 5, Table 1), indicated that the early heading phenotype in *eh1* is not due to mutation of large deletion in *VRN-B1*. In addition, due to the deletion in the first intron of *VRN-B1*, the *eh1* and WT was able to flower without vernalization (Table 1, Fig. 5). Since the variations in WT and *eh1* at the position of primer Intrl/B/F [16] leaded to failed amplification (Fig. S3A, B), the new markers developed in this study would be an alternative solution for marker-assisted breeding in wheat. The sequencing of *VRN-B1* gene suggested that a 37 bp deletion located downstream of the large deletion was mutated in *eh1* (Fig. S3A, C) and this 37 bp deletion co-segregated with the large deletion in the RIL population. The 37 bp deletion is similar to a 36 bp deletion region of previously reported *Vrn-B1b*, which was present in the spring wheat cultivar [18]. It has been reported that accessions containing the *Vrn-B1b* allele exhibited longer days to heading than those carrying the *Vrn-B1a* alleles [36]. In contrast, by phenotype analysis of F_2_ individuals derived from cross of *eh1* and WT, we found that the 37 bp deletion didn’t result in HD variation (Fig. S4). Since only 2 accessions carrying *Vrn-B1b* allele were used for comparison by Cho et al. [36], it is possible that the background differences of other loci affecting heading time among various accessions leaded to the detection of HD variation.

Due to the limited lines used for BSR-Seq, only one QTL located on chromosome 5B was detected by using this approach (Fig. 2). When we used different early heading or late heading lines to construct three pairs of bulks for BSA analysis, QTLs located on chromosomes 5B, 2A, 2B, 3A, 3B and 6A were detected (Fig. S2A-C). The QTL on chromosome 5B detected by three pairs of bulks also included the *VRN-B1* gene (Fig. S2D), which is in consistent with the results from BSR-Seq and genetic mapping (Figs. 2, 3). Previous studies also found QTLs associated with HD located on chromosomes 5B, 2A, 2B, 3A and 3B by genetic mapping [37–39] and the flowering regulator *photoperiod* genes *Ppd-A1* and *Ppd-B1* in wheat are located on chromosomes 2A and 2B, respectively [40]. Although a 131 bp deletion on the 3′ region of *Ppd-A1* gene was detected in *eh1* compared to LX987 (Fig. S5C), the locus detected on chromosome 2A was not *Ppd-A1* gene due to no difference of heading date between RILs with and without this deletion (Fig. S5D). Additionally, genome-wide association analysis also detected the QTL for HD on chromosome 6A [41, 42]. It is possible that the genetic background diversity for HD between WT and LX987 leads to detection of several QTLs in the RIL population. Since different RILs contain distinct genes for HD, using different RILs for BSA analysis would be a feasible and comprehensive approach for QTL identification in such population. However, since natural variations for HD existed between *eh1* and LX987, it is difficult to distinguish the mutation loci from these QTLs. This problem would be solved by further fine mapping or using the population derived from cross of mutant and WT.

Together, several studies have elucidated the genetic and phenotypic effects of different variations in the *VRN1* locus over the past several decades. However, the impact of *VRN1* deletions on global gene expression has not been widely reported [42]. Using BSR-Seq data, differentially expressed genes (DEGs) in young spikes were identified between two pools comprised of those with either the *Vrn-B1* or *vrn-B1* genes (Table S4). Although the DEGs detected in the RNAseq analysis might not be solely due to the large deletion in *VRN-B1*, the newly discovered genes and pathways by expression pattern analysis would be partly regulated by *VRN1* or affected by different flowering time. One limitation is un-replicated data for RNA-seq and it would be partly offset by analysis of the overlapped DEGs between parents and bulks. It has been reported that the Vrn1 protein binds to the promoter of *FT1*/ *Vrn3* by chromatin immunoprecipitation sequencing (ChIP-seq), and transcript levels of *FT1*/ *Vrn3* were upregulated by high expression of *Vrn1* in barley [43]. *VRN1* also down-regulates the flowering repressor *VRN2* gene [24]. Consistently, higher expression levels of *VRN-A1*, *VRN-B1* and *VRN-D1* in the spikes of *eh1* compared to that of LX987 were detected while lower amounts of *VRN2* were observed in *eh1* (Fig. S6). Meanwhile, increased expression of *Vrn-B3* was detected in *eh1*, or early heading bulks that had the *Vrn-B1* genotype relative to those within the *vrn-B1* group, suggesting *Vrn-B1* may play a role in regulating *Vrn-B3* in wheat. Genes that potentially involved in floral development including CONSTANS-like genes, MADS-box genes and MYB transcription factors were identified in our analysis (Table S5). These genes were also identified as VRN1-binding targets that potentially regulate flowering through RNA-Seq and ChIP-seq by Deng et al. [43]. Furthermore, extremely up-regulated genes with a fold change > 100, including Beta-amylase (*BMY1*) and anther-specific protein (*RTS*), may also be regulated by *Vrn-B1* and play a key role in the relationship between heading and flowering. Additionally, functional enrichment analyses also indicated that there are several significantly enriched pathways among the DEGs, such as carbon metabolism, starch and sucrose metabolism, glycolysis, gluconeogenesis, and fatty acid biosynthesis and metabolism (Table S7). Deng et al. identified direct targets of *VRN1* including genes involved in the biosynthesis or breakdown of jasmonic acid, abscisic acid and gibberellin [43]. Similarly, these pathways, except for gibberellin, were enriched in our GO analysis (Table S6), indicating these pathways are conserved in regulating flowering of cereals. Carbohydrates play an important role in the control of flowering transition in plants. The accumulation of sucrose, a major sugar in both leaf and apical exudates, in the meristem precedes the activation of energy-consuming processes such as mitotic activation [44, 45]. The *Arabidopsis* late flowering mutant *carbohydrate accumulation1* (*cam1*) contains higher starch levels than WT at the onset of flowering, but the late flowering phenotype is not caused by increased starch levels [46]. Interestingly, this study suggested that flowing time had an effect on expression patterns of the genes directly or indirectly participating in carbohydrate metabolism. Fatty acid metabolism is important for pollen development. The cell walls of pollen contain fatty substances produced in the tapetum of anthers [47]. In addition, the early flowering phenotype caused by overexpression of fatty acid amide hydrolase in *Arabidopsis* also supports the observation that fatty acids play an important role in floral induction [48]. Consequently, we observed that DEGs involved in fatty acid biosynthesis and metabolism were enriched in lines with flowering time variants. It also indicated that fatty acid metabolic pathways interact with the flower development pathway in wheat and may be affected by variations in *VRN-B1* gene expression. The flavonoid biosynthesis pathway has been well documented and is known to be associated with flowering time variation in plants [49–51]. Flavonoids are a class of powerful antioxidant compounds whose synthesis is initiated by the phenylpropanoid pathway and subsequently routes into nine different metabolic branches, including those that produce flavones and flavonols [52]. Interestingly, our transcriptomic analysis indicated that pathways related to phenylpropanoid biosynthesis, phenylalanine metabolism, flavonoid biosynthesis, flavone biosynthesis, and flavonol biosynthesis were significantly enriched in our data set (Table S7) and further detailed analysis of DEGs for phenylpropanoid biosynthesis suggested that 98 up-regulated-genes and 16 down-regulated genes participating in most of steps in the pathway were identified (Fig. S8), indirectly suggesting that these pathways are involved in the early heading process or influenced by flowering time in wheat.

## Conclusions

In the present study, a major QTL for heading time was identified in a wheat RIL population using a combination of BSA and genetic mapping. Development of molecular markers for *Vrn-B1* suggested that a large deletion in the first intron was partially responsible for heading time variation in the RIL population. Additionally, our results also revealed several important genes and pathways associated with flowering. The molecular markers for *VRN-B1* developed in this study will be deployed for marker assisted breeding programs to optimize heading time, and the candidate DEGs associated with *Vrn-B1* gene regulation help us to better understand the molecular function of heading-related pathways in wheat.

## Methods

### Plant materials and phenotypic analysis

The early heading mutant (*eh1*) was obtained by mutagenesis of dry seeds of spring wheat line Zhongyuan9 with 284 Gy γ-ray irradiation. The *eh1* mutant was crossed with a late heading Chinese elite cultivar, Lunxuan987 (LX987), to produce F_6:7_ recombinant inbred lines (RILs) by single-seed descent. Zhongyuan9 wheat lines and the *eh1* were created in our lab, and the seeds of LX987 were kindly provided by Binghua Liu (Institute of Crop Sciences, Chinese Academy of Agricultural Sciences). The RIL population (400 lines) and parent lines were sown at the Zhongpuchang station of the Institute of Crop Sciences, Chinese Academy of Agricultural Sciences (Beijing, China) and grown under well-managed field conditions. Each line was planted with 15 plants in a row of 1 m. The heading time of each RIL was recorded from 3 years’ experiment of 2016 to 2018 when two thirds of the plants in a line were headed and more than half of the spikes had emerged.

### BSR-Seq analysis

When the early heading RILs were beginning to heading, young spikes from extremely early and late heading RILs, and two parents were flash frozen in liquid nitrogen and then stored at − 80 °C. In total, 21 early heading and 22 late heading lines were used. RNA was extracted from these lines using TRNzol-A^+^ Reagent (Tiangen Biotech, China). RNA samples were treated with DNase I (Takara) and cleaned using an RNA purification kit (Tiangen Biotech). An equal amount of RNA from each line was mixed to construct early and late heading bulks. The RNA quality was assessed using both a NanoDrop™ One (Thermo Scientific) and a Bioanalyzer 2100 system (Agilent Technologies). A total of 4 cDNA libraries, including two parents and two bulks, were successfully constructed using the NEBNext® Ultra™ RNA Library Prep Kit for Illumina® (NEB). RNA sequencing was carried out on an Illumina HiSeq platform according to the manufacturer’s protocol and 150 bp paired-end reads were generated (Biomarker Technologies Corporation, Beijing). After removing low-quality reads, reads with adapters or those with more than 10% unidentified nucleotides from raw data, a total of 54.71 Gb of clean reads were obtained. The detailed information of number of reads per library, Q30 and GC content were shown in Table S8. The clean reads were then aligned to the reference genome Chinese Spring wheat v1.0 released by the International Wheat Genome Sequencing Consortium (IWGSC) (http://www.wheatgenome.org/) using STAR software with default parameters [53]. Duplicates were excluded based on their position in the reference genome using Picard (https://sourceforge.net/projects/picard/). Local realignments, base recalibration, and SNP calling was conducted using GATK software [54]. High-quality SNPs were obtained according to the following filtering criteria: (i) loci were removed if multiple alleles exist; (ii) SNP loci with reads support < 4 were filtered out; (iii) SNPs with the same genotype in bulks were excluded; (iv) SNPs from late heading bulks that differed from the late heading parent were discarded. The sequencing datasets generated in this study are deposited in the National Centre for Biotechnology Information (NCBI) under the BioProject ID PRJNA517367 with the Sequence Read Achieve (SRA) accession SRP182626.

### Association analysis of ED and SNP-index

The Euclidean Distance (ED) algorithm evaluates the correlation between the target region and the trait of interest based on the significant difference of markers from sequencing data in bulks [55]. Theoretically, markers close to the target region are different in two extreme phenotype bulks while others out of the region are consistent, and their ED value is close to 0. The higher the ED value is, the greater the difference between two bulks. The formula for ED is as follows:

$$ ED=\sqrt{{\left({A}_{mut}-{\mathrm{A}}_{WT}\right)}^2+{\left({C}_{mut}-{\mathrm{C}}_{WT}\right)}^2+{\left({G}_{mut}-{\mathrm{G}}_{WT}\right)}^2+{\left({T}_{mut}-{\mathrm{T}}_{WT}\right)}^2} $$

X_mut_, X_WT_ represents the frequency of X base in mutant and wild type bulk, respectively. In the analysis, the sequencing depth and ED value were calculated for each different SNP in the bulks. To eliminate the background noise, the fifth power of the original ED value was regarded as a correlation value and then the ED value was fitted using the SNPNUM algorithm. The SNP-index was defined as the ratio between mutant allele frequency and wild type allele frequency in bulks [56, 57]. The index is equal to 1 when all SNP reads differ from the wild type allele, and is equal to 0 when all SNP reads are identical to the wild type allele. To exclude the false positives, SNPs located on the same chromosome were fitted according to their position in the genome. The regions above the association threshold were considered as the candidate interval related to the early heading phenotype. The number of genes in the candidate region was calculated according to the number of predicted genes in the corresponding region of reference genome.

### Genomic DNA extraction

Genomic DNA was extracted from fresh leaf samples at the seedling stage as previously described [58]. Briefly, after grinding the frozen leaves into homogeneous powder by using a Vibration Mill Type MM301 (Restsch GmbH, Germany), 600 μL DNA extraction buffer was added and completely mixed. The samples were then incubated at 65 °C for 1 h. Following the addition of 200 μL 5 mol∙L^− 1^ KAc, a 300 μL aliquot of the supernatant was collected after centrifugation. DNA sample was precipitated by isopropanol and then washed with 70% ethanol. The quality and quantity of DNA were determined using a NanoDrop ND-2000 Spectrophotometer (Thermo Scientific), and the final concentration was normalized to 60 ng∙μL^− 1^.

### Bulked Segregant analysis (BSA) based on wheat 660 K SNP array

Based on 2 years phenotype data, the extremely early heading or late heading RILs were selected. Three early heading or late heading bulks were constructed by mixing the same amount of DNA from each RILs. Specifically, the early heading bulk 1 and bulk 2 were mixed with 18 early heading RILs, respectively. The early heading bulk 3 was mixed with 22 lines and the three late heading bulks were mixed with 16 lines, respectively. A total of 6 bulks and 2 parents were genotyped using the Axiom® wheat 660 K SNP array (Thermo). High quality genotyping data was obtained by using the threshold of DQC (Dish QC) > 0.82 and CR (Call-Rate) > 94. The SNPs showing different genotypes in a pair of early and late heading bulks, and the same genotypes between bulk and parent with the same phenotype, were selected. Finally, the number of selected SNP distributed on each chromosome was calculated. The chromosome with higher number of selected SNP was considered as the QTL associated with HD. SNP frequency across the chromosome was determined by the number of SNP in a 10-Mb region divided by the total number of SNP in the chromosome.

### Development of Kompetitive allele specific PCR (KASP) markers

Based on the RNA-seq data, the SNP on candidate chromosomes were selected and converted to Kompetitive Allele Specific PCR (KASP) markers using the online primer design pipeline PolyMarker (http://polymarker.tgac.ac.uk/). The specificity of KASP markers were tested using two parent lines. PCR assays were conducted using a CFX 96 Real-Time System (Bio Rad, Hercules, CA, USA). A 5 μL reaction, including 2.5 μL KASP master mixture (LGC Genomics, Middlesex, UK), 2.4 μL 60 ng∙μL^− 1^ DNA, 0.04 μL 50 mM Mg^2+^ and 0.06 μL primer mix (primer A (100 μM): primer B (100 μM): primer R (100 μM): ddH_2_O = 12: 12: 30: 46), were performed for PCR with the following procedures: 95 °C for 15 min, followed by 10 cycles of touch-down (95 °C for 20 s; touchdown at 65 °C initially and decreasing by 0.5 °C per cycle for 30 s), then followed by 30 additional cycles of annealing (95 °C for 10 s; 57 °C for 60 s).

### Construction of a genetic linkage map

A linkage map of the candidate chromosome was constructed using QTL IciMapping version 4.1 [59, 60]. Recombination frequency was converted into centimorgan (cM) distances with the Kosambi map function [61]. QTL mapping was conducted using the days to heading in three individual years with the inclusive composite interval mapping (ICIM) analysis. A value of phenotypic variance explained (*PVE*) by an individual QTL was determined using ICIM. Significant QTLs were identified at a logarithm of odds (LOD) threshold of 3.0.

### Identification of *VRN-B1*

Two pairs of primers were designed to identify the presence or absence of a large deletion in *VRN-B1*. The forward and reverse primer pair termed TavBI were designed to flank the deletion region so that product could only be detected in the dominant *Vrn-B1*. For the primer pair termed TavBII, the forward primer was designed to anneal to the deletion region and the reverse primer was designed to anneal to a region of the genome outside of the deletion so that product can only be detected in the recessive *vrn-B1*. PCR reactions were performed using T_5_ Super PCR mix (Tsingke, Beijing) with the following procedure: 98 °C for 3 min, followed by 32 cycles of annealing (98 °C for 10 s, 58 °C for 10 s; 72 °C for 1 min.). The ratio of dominant effect that represent the difference of heading time between heterozygote and the midpoint of two parents, and additive effect that represent the half value of the difference of two parents, was considered to be the degree of dominance. For sequencing *Vrn-B1*, 11 pairs of primers were designed to amplify the whole gene and then sequenced. The primers for sequencing of *VRN-B1* and *Ppd-A1* and marker primers for detection of a 37 bp deletion in *VRN-B1* and a 131 bp deletion in *Ppd-A1* are shown in Table S9.

### Quantification of gene expression levels and DEGs analysis

Based on the BSR-Seq data, the number of clean reads mapped to each gene was counted using the Cuffquant and Cuffnorm modules in Cuffinks software. The expected gene expression levels from two parents and two bulks were calculated using Per Kilobase of transcript per Million fragments mapped (FPKM). The FPKM algorithm normalizes transcript lengths and the number of mapped reads, and is a commonly used method for estimating gene expression levels [62]. Differentially expressed genes (DEGs) analysis was performed using the EBSeq R package, which provides a statistical method based on an empirical Bayes hierarchical model. The resulting *P*-value was adjusted using the Benjamini-Hochberg approach in order to control the false discovery rate. Genes were considered to be differentially expressed between two groups if the adjusted *P*-values were < 0.05 (FDR < 0.05) by EBSeq and the Fold Change ≥2.0.

### GO term and KEGG enrichment analysis

Gene Ontology (GO; http://www.geneontology.org) enrichment analysis of DEGs was performed using the GOseq R package (corrected *P*-value < 0.05), in which gene length bias was corrected. GO terms with corrected *P*-values less than 0.05 were considered significantly enriched. Based on the retrieving of KEGG pathways for DEGs (http://www.genome.jp/kegg/), the statistical significance of the enrichment of DEGs in each KEGG pathway was analyzed by using KOBAS software. KEGG mapper was used for analysis of the detailed DEGs in the most significantly enriched pathway (https://www.kegg.jp/kegg/mapper.html).

### Quantitative real-time PCR analysis

The same tissues as used in RNAseq analysis (*eh1* and LX987 spike samples) were used for quantitative real-time PCR verification. For quantifying the relative expression of vernalization genes, young spikes and leaves of *eh1* and LX987 were sampled when *eh1* started to heading. For analysis of the gene expression at the same developmental stage, young spikes and leaves of *eh1* were also sampled 8 days earlier than that of LX987 when they showed similar developmental levels of spikes. RNA extraction and qRT-PCR were conducted as previously reported [63]. Generally, total RNA from spike or leaf sample was isolated using an RNeasy Plant Mini Kit (Qiagen). DNase I (Takara) and RNA purification kit (Tiangen) were used for elimination any DNA contamination in the extracted RNA. By using a TransScript First-Strand cDNA Synthesis SuperMix (TransGen) kit, the first strand cDNA was synthesized. The qRT-PCR was performed using the SsoFast EvaGreen Supermix Kit (Bio-Rad, USA) and a CFX 96 Real-TimeSystem (Bio-Rad, USA). This experiment was conducted with two technical repeats and three independent biological replicates. For verification of DEGs, gene-specific primers were designed using Oligo 7 software. The details of primers were listed on Table S10. For expression analysis of *VRN1* and *VRN2* genes, primers described in previous studies [7, 64] were used. *ACTIN* was used as an internal control to normalize the expression data. Relative expression levels were determined using the 2 ^–ΔΔCT^ method [65].

## Supplementary information

**Additional file 1: Fig S1.** Distribution of heading date in the RIL population in 2016, 2017 and 2018.

**Additional file 2: Fig S2.** QTLs for HD in the RIL population detected by BSA analysis. (A-C) The number of SNP associated with HD on each chromosome between the early and late heading bulk 1 (A), bulk 2 (B), and bulk 3 (C). The QTL is present on chromosomes enriched higher number of selected SNP. (D) The frequency of SNP associated with HD distributed on chromosome 5B. The blue, red, and green lines represent bulk 1, bulk 2, and bulk 3, respectively.

**Additional file 3: Fig S3.** Sequence comparison of *VRN-B1* in LX987, WT, and *eh1*. (A) Schematic representation of *VRN-B1* in LX987, WT, and *eh1*. (B) Sequence analysis of primer Intrl/B/F in LX987, WT, and *eh1*. (C) Polymerase chain reaction amplification of a 37 bp deletion in *eh1* by using specific primers. Full-length gels are presented in Supplementary Figure 10.

**Additional file 4: Fig S4.** Days to heading of F_2_ individuals with or without 37 bp deletion of *VRN-B1*. WT indicates without 37 bp deletion while *eh1* indicates with 37 bp deletion.

**Additional file 5: Fig S5.** Variations of *VRN-A1* and *Ppd-A1* between LX987 and *eh1*. (A) Identification of natural variation of *VRN-A1* in LX987 and *eh1* by previously developed markers. Full-length gels are presented in Supplementary Figure 11. (B) Identification of the 131 bp deletion of *Ppd-A1* in *eh1* and Chinese Spring (CS). Full-length gels are presented in Supplementary Figure 12. (C) Sequence comparison of *Ppd-A1* in LX987, WT, and *eh1*. (D) Days to heading of RILs with or without 131 bp deletion of *Ppd-A1*.

**Additional file 6: Fig S6.** Relative expression levels of *VRN-A1*, *VRN-B1*, *VRN-D1*, and *VRN2* genes in the young spikes and leaves of *eh1* and LX987 when sampling at the same day. Student’s t-tests were used to assess the significance. *P < 0.05.

**Additional file 7: Fig S7.** Relative expression levels of *VRN-A1*, *VRN-B1*, *VRN-D1*, and *VRN2* genes in the young spikes and leaves of *eh1* and LX987 when sampling at the same developmental stage. Student’s t-tests were used to assess the significance. **P < 0.01 and *P < 0.05.

**Additional file 8: Fig S8.** DEGs in phenylpropanoid biosynthesis. The red box filled with blue color indicates the up-regulated genes in the early heading bulk and the parent *eh1*, the black box filled with blue color represents down-regulated genes, and the box without color is genes showing no expression difference.

**Additional file 9: Fig S9.** Original gel image of Fig. 4a.

**Additional file 10: Fig S10.** Original gel image of Fig S3C.

**Additional file 11: Fig S11.** Original gel image of Fig S5A.

**Additional file 12: Fig S12.** Original gel image of Fig S5B.

**Additional file 13: Table S1.** HD variation in RILs, WT and parent lines.

**Additional file 14: Table S2.** Heading date of RIL lines in 2016, 2017 and 2018.

**Additional file 15: Table S3.** Primers used for mapping and variant identification.

**Additional file 16: Table S4.** Genotype and days to heading of RILs used in RNA pooling.

**Additional file 17: Table S5.** Differentially expressed genes in parents and heading pools.

**Additional file 18: Table S6.** Enriched GO terms of overlapped DEGs.

**Additional file 19: Table S7.** Enriched KEGG pathways of overlapped DEGs.

**Additional file 20: Table S8.** Summary of the sequencing data generated in this study.

**Additional file 21: Table S9.** Primers for sequencing of *VRN-B1* and marker primers for detection of a 37 bp deletion in *VRN-B1*.

**Additional file 22: Table S10.** Primers used for RT-qPCR.

## Data Availability

The data supporting the conclusions of this study are within the paper and its additional files. All sequencing datasets are deposited in the National Centre for Biotechnology Information (NCBI) under the BioProject ID PRJNA517367 with the Sequence Read Achieve (SRA) accession SRP182626.

## Abbreviations

RILs: Recombinant inbred lines
eh1: Early heading mutant 1
BSA: Bulked Segregant Analysis
QTL: Quantitative trait loci
BMY1: Beta-amylase 1
RTS: Anther-specific protein
DEGs: Differentially expressed genes
FT: FLOWERING LOCUS T
CAL: CAULIFLOWER
AP1: APETALA1
FUL: FURITFULL
RIP3: RNA immune precipitation fragment 3
bZIP: Basic leucine-zipper
NF-Y: Nuclear factor-Y
HD: Heading date
BSR-Seq: Bulked Segregant RNA-Seq
ED: Euclidean Distance
GO: Gene ontology
KEGG: Kyoto Encyclopedia of Genes and Genomes

## References

[1] Jung C, Muller AE (2009). Flowering time control and applications in plant breeding. Trends Plant Sci.

[2] Chouard P (1960). Vernalization and its relations to dormancy. Annu Rev Plant Physiol.

[3] Yan L, Loukoianov A, Tranquilli G, Helguera M, Fahima T, Dubcovsky J (2003). Positional cloning of the wheat vernalization gene *VRN1*. Proc Natl Acad Sci U S A.

[4] Yan L, Loukoianov A, Blechl A, Tranquilli G, Ramakrishna W, SanMiguel P, Bennetzen J, Echenique V, Dubcovsky J (2004). The wheat *VRN2* gene is a flowering repressor down-regulated by vernalization. Science.

[5] Yan L, Fu D, Li C, Blechl A, Tranquilli G, Bonafede M, Sanchez A, Valarik M, Yasuda S, Dubcovsky J (2006). The wheat and barley vernalization gene *VRN3* is an orthologue of FT. Proc Natl Acad Sci U S A.

[6] Kippes N, Debernardi JM, Vasquez-Gross HA, Akpinar BA, Budak H, Kato K, Chao S, Akhunov E, Dubcovsky J (2015). Identification of the *VERNALIZATION 4* gene reveals the origin of spring growth habit in ancient wheats from South Asia. Proc Natl Acad Sci U S A.

[7] Dubcovsky J, Loukoianov A, Fu D, Valarik M, Sanchez A, Yan L (2006). Effect of photoperiod on the regulation of wheat vernalization genes *VRN1* and *VRN2*. Plant Mol Biol.

[8] Distelfeld A, Li C, Dubcovsky J (2009). Regulation of flowering in temperate cereals. Curr Opin Plant Biol.

[9] Dubcovsky J, Chen C, Yan L (2005). Molecular characterization of the allelic variation at the *VRN-H2* vernalization locus in barley. Mol Breed.

[10] Li Y, Xu M (2017). CCT family genes in cereal crops: a current overview. Crop J.

[11] Ferrándiz C, Gu Q, Martienssen R, Yanofsky MF (2000). Redundant regulation of meristem identity and plant architecture by *FRUITFULL*, *APETALA1* and *CAULIFLOWER*. Development.

[12] Dhillon T, Pearce SP, Stockinger EJ, Distelfeld A, Li C, Knox AK, Vashegyi I, Vagujfalvi A, Galiba G, Dubcovsky J (2010). Regulation of freezing tolerance and flowering in temperate cereals: the *VRN-1* connection. Plant Physiol.

[13] Preston JC, Kellogg EA (2006). Reconstructing the evolutionary history of paralogous APETALA1/FRUITFULL-like genes in grasses (Poaceae). Genetics.

[14] Golovnina KA, Kondratenko EY, Blinov AG, Goncharov NP (2010). Molecular characterization of vernalization loci *VRN1* in wild and cultivated wheats. BMC Plant Biol.

[15] Yan L, Helguera M, Kato K, Fukuyama S, Sherman J, Dubcovsky J (2004). Allelic variation at the *VRN-1* promoter region in polyploid wheat. Theor Appl Genet.

[16] Fu D, Szucs P, Yan L, Helguera M, Skinner JS, von Zitzewitz J, Hayes PM, Dubcovsky J (2005). Large deletions within the first intron in *VRN-1* are associated with spring growth habit in barley and wheat. Mol Gen Genomics.

[17] Kippes N, Guedira M, Lin L, Alvarez MA, Brown-Guedira GL, Dubcovsky J (2018). Single nucleotide polymorphisms in a regulatory site of *VRN-A1* first intron are associated with differences in vernalization requirement in winter wheat. Mol Gen Genomics.

[18] Santra DK, Santra M, Allan RE, Campbell KG, Kidwell KK (2009). Genetic and molecular characterization of vernalization genes *Vrn-A1*, *Vrn-B1*, and *Vrn-D1* in spring wheat germplasm from the Pacific northwest region of the USA. Plant Breed.

[19] Chu CG, Tan CT, Yu GT, Zhong S, Xu SS, Yan L (2011). A novel retrotransposon inserted in the dominant *Vrn-B1* allele confers spring growth habit in Tetraploid Wheat (*Triticum turgidum L.*). G3 (Bethesda).

[20] Milec Z, Tomkova L, Sumikova T, Pankova K (2012). A new multiplex PCR test for the determination of *Vrn-B1* alleles in bread wheat (*Triticum aestivum L.*). Mol Breed.

[21] Shcherban AB, Efremova TT, Salina EA (2012). Identification of a new *Vrn-B1* allele using two near-isogenic wheat lines with difference in heading time. Mol Breed.

[22] Zhang B, Wang X, Wang X, Ma L, Wang Z, Zhang X (2018). Molecular characterization of a novel vernalization allele *Vrn-B1d* and its effect on heading time in Chinese wheat (*Triticum aestivum L.*) landrace Hongchunmai. Mol Breed.

[23] Hemming MN, Peacock WJ, Dennis ES, Trevaskis B (2008). Low-temperature and daylength cues are integrated to regulate *FLOWERING LOCUS T* in barley. Plant Physiol.

[24] Chen A, Dubcovsky J (2012). Wheat TILLING mutants show that the vernalization gene *VRN1* down-regulates the flowering repressor *VRN2* in leaves but is not essential for flowering. PLoS Genet.

[25] Distelfeld A, Tranquilli G, Li C, Yan L, Dubcovsky J (2009). Genetic and molecular characterization of the *VRN2* loci in tetraploid wheat. Plant Physiol.

[26] Li C, Dubcovsky J (2008). Wheat FT protein regulates *VRN1* transcription through interactions with FDL2. Plant J.

[27] Abe M, Kobayashi Y, Yamamoto S, Daimon Y, Yamaguchi A, Ikeda Y, Ichinoki H, Notaguchi M, Goto K, Araki T (2005). FD, a bZIP protein mediating signals from the floral pathway integrator FT at the shoot apex. Science.

[28] Li C, Distelfeld A, Comis A, Dubcovsky J (2011). Wheat flowering repressor VRN2 and promoter CO2 compete for interactions with NUCLEAR FACTOR-Y complexes. Plant J.

[29] Milec Z, Valarik M, Bartos J, Safar J (2014). Can a late bloomer become an early bird? Tools for flowering time adjustment. Biotechnol Adv.

[30] Kiseleva AA, Salina EA (2018). Genetic regulation of common wheat heading time. Russ J Genet.

[31] Liu S, Yeh CT, Tang HM, Nettleton D, Schnable PS (2012). Gene mapping via bulked segregant RNA-Seq (BSR-Seq). PLoS One.

[32] Ramirez-Gonzalez RH, Uauy C, Caccamo M (2015). PolyMarker: a fast polyploid primer design pipeline. Bioinformatics.

[33] Langer SM, Longinand CFH, Wuerschum T (2014). Flowering time control in European winter wheat. Front Plant Sci.

[34] Nawaz Z, Shu Q (2014). Molecular nature of chemically and physically induced mutants in plants: a review. Plant Genet Resour Charact Util.

[35] von Zitzewitz J, Szucs P, Dubcovsky J, Yan L, Francia E, Pecchioni N, Casas A, Chen TH, Hayes PM, Skinner JS (2005). Molecular and structural characterization of barley vernalization genes. Plant Mol Biol.

[36] Cho E, Kang C, Jung J, Yoon Y, Park C (2015). Allelic variation of Rht-1, Vrn-1 and Ppd-1 in Korean wheats and its effect on agronomic traits. Plant Breed Biotech.

[37] Luo W, Ma J, Zhou XH, Sun M, Kong XC, Wei YM, Jiang YF, Qi PF, Jiang QT, Liu YX (2016). Identification of quantitative trait loci controlling agronomic traits indicates breeding potential of Tibetan semiwild wheat (*Triticum aestivum ssp. tibetanum*). Crop Sci.

[38] Li F, Wen W, He Z, Liu J, Jin H, Cao S, Geng H, Yan J, Zhang P, Wan Y (2018). Genome-wide linkage mapping of yield-related traits in three Chinese bread wheat populations using high-density SNP markers. Theor Appl Genet.

[39] Enid PL, Semagn K, Chen H, Iqbal M, N'Diaye A, Kamran A, Navabi A, Pozniak C, Spaner D (2016). QTLs associated with agronomic traits in the cutler x AC Barrie spring wheat mapping population using single nucleotide polymorphic markers. PLoS One.

[40] Snape JW, Butterworth K, Whitechurch E, Worland AJ (2001). Waiting for fine times: genetics of flowering time in wheat. Euphytica.

[41] Le Gouis J, Bordes J, Ravel C, Heumez E, Faure S, Praud S, Galic N, Remoue C, Balfourier F, Allard V (2012). Genome-wide association analysis to identify chromosomal regions determining components of earliness in wheat. Theor Appl Genet.

[42] Diallo AO, Agharbaoui Z, Badawi MA, Ali-Benali MA, Moheb A, Houde M, Sarhan F (2014). Transcriptome analysis of an *mvp* mutant reveals important changes in global gene expression and a role for methyl jasmonate in vernalization and flowering in wheat. J Exp Bot.

[43] Deng W, Casao MC, Wang P, Sato K, Hayes PM, Finnegan EJ, Trevaskis B (2015). Direct links between the vernalization response and other key traits of cereal crops. Nat Commun.

[44] Georges B, Andree H, Claude H, Anne P, Pierre L (1993). Physiological signals that induce flowering. Plant Cell.

[45] Corbesier L, Lejeune P, Bernier G (1998). The role of carbohydrates in the induction of flowering in Arabidopsis thaliana: comparison between the wild type and a starchless mutant. Planta.

[46] Eimert K, Wang SM, Lue WL, Chen J (1995). Monogenic recessive mutations causing 60th late floral initiation and excess starch accumulation in Arabidopsis. Plant Cell.

[47] Blackmore S, Wortley AH, Skvarla JJ, Rowley JR (2007). Pollen wall development in flowering plants. New Phytol.

[48] Teaster ND, Keereetaweep J, Kilaru A, Wang YS, Tang Y, Tran CN, Ayre BG, Chapman KD, Blancaflor EB (2012). Overexpression of fatty acid amide hydrolase induces early flowering in *Arabidopsis thaliana*. Front Plant Sci.

[49] Kumar V, Nadda G, Kumar S, Yadav SK (2013). Transgenic tobacco overexpressing tea cDNA encoding dihydroflavonol 4-Reductase and Anthocyanidin Reductase induces early flowering and provides biotic stress tolerance. PLoS One.

[50] Yao QY, Huang H, Tong Y, Xia EH, Gao LZ (2016). Transcriptome analysis identifies candidate genes related to triacylglycerol and pigment biosynthesis and photoperiodic flowering in the ornamental and oil-producing plant, *Camellia reticulata* (Camellia). Front Plant Sci.

[51] Yue J, Zhu C, Zhou Y, Niu X, Miao M, Tang X, Chen F, Zhao W, Liu Y (2018). Transcriptome analysis of differentially expressed unigenes involved in flavonoid biosynthesis during flower development of Chrysanthemum morifolium 'Chuju'. Sci Rep.

[52] Winkel-Shirley B (2001). Flavonoid biosynthesis. A colorful model for genetics, biochemistry, cell biology, and biotechnology. Plant Physiol.

[53] Dobin A, Davis CA, Schlesinger F, Drenkow J, Zaleski C, Jha S, Batut P, Chaisson M, Gingeras TR (2013). STAR: ultrafast universal RNA-seq aligner. Bioinformatics.

[54] McKenna A, Hanna M, Banks E, Sivachenko A, Cibulskis K, Kernytsky A, Garimella K, Altshuler D, Gabriel S, Daly M (2010). The genome analysis toolkit: a MapReduce framework for analyzing next-generation DNA sequencing data. Genome Res.

[55] Hill JT, Demarest BL, Bisgrove BW, Gorsi B, Su YC, Yost HJ (2013). MMAPPR: mutation mapping analysis pipeline for pooled RNA-seq. Genome Res.

[56] Abe A, Kosugi S, Yoshida K, Natsume S, Takagi H, Kanzaki H, Matsumura H, Yoshida K, Mitsuoka C, Tamiru M (2012). Genome sequencing reveals agronomically important loci in rice using MutMap. Nat Biotechnol.

[57] Takagi H, Abe A, Yoshida K, Kosugi S, Natsume S, Mitsuoka C, Uemura A, Utsushi H, Tamiru M, Takuno S (2013). QTL-seq: rapid mapping of quantitative trait loci in rice by whole genome resequencing of DNA from two bulked populations. Plant J.

[58] Li W, Guo H, Wang Y, Xie Y, Zhao L, Gu J, Zhao S, Zhao B, Wang G, Liu L (2017). Identification of novel alleles induced by EMS-mutagenesis in key genes of kernel hardness and starch biosynthesis in wheat by TILLING. Genes Genom.

[59] Li H, Ye G, Wang J (2007). A modified algorithm for the improvement of composite interval mapping. Genetics.

[60] Meng L, Li H, Zhang L, Wang J (2015). QTL IciMapping: integrated software for genetic linkage map construction and quantitative trait locus mapping in biparental populations. Crop J.

[61] Kosambi DD (1943). The estimation of map distances from recombination values. Ann Eugenics.

[62] Trapnell C, Williams BA, Pertea G, Mortazavi A, Kwan G, van Baren MJ, Salzberg SL, Wold BJ, Pachter L (2010). Transcript assembly and quantification by RNA-Seq reveals unannotated transcripts and isoform switching during cell differentiation. Nat Biotechnol.

[63] Xiong H, Guo H, Xie Y, Zhao L, Gu J, Zhao S, Li J, Liu L (2017). RNAseq analysis reveals pathways and candidate genes associated with salinity tolerance in a spaceflight-induced wheat mutant. Sci Rep.

[64] Loukoianov A, Yan L, Blechl A, Sanchez A, Dubcovsky J (2005). Regulation of *VRN-1* vernalization genes in normal and transgenic polyploid wheat. Plant Physiol.

[65] Livak KJ, Schmittgen TD (2001). Analysis of relative gene expression data using real-time quantitative PCR and the 2^-ΔΔCT^ method. Methods.

