# Supplementary figures and images for: Identification of the vernalization gene VRN-B1 responsible for heading date variation by QTL mapping using a RIL population in wheat
Source: BMC Plant Biol. 2020 Jul 13;20:331. doi: 10.1186/s12870-020-02539-5 (PMC7359472; doi:10.1186/s12870-020-02539-5)

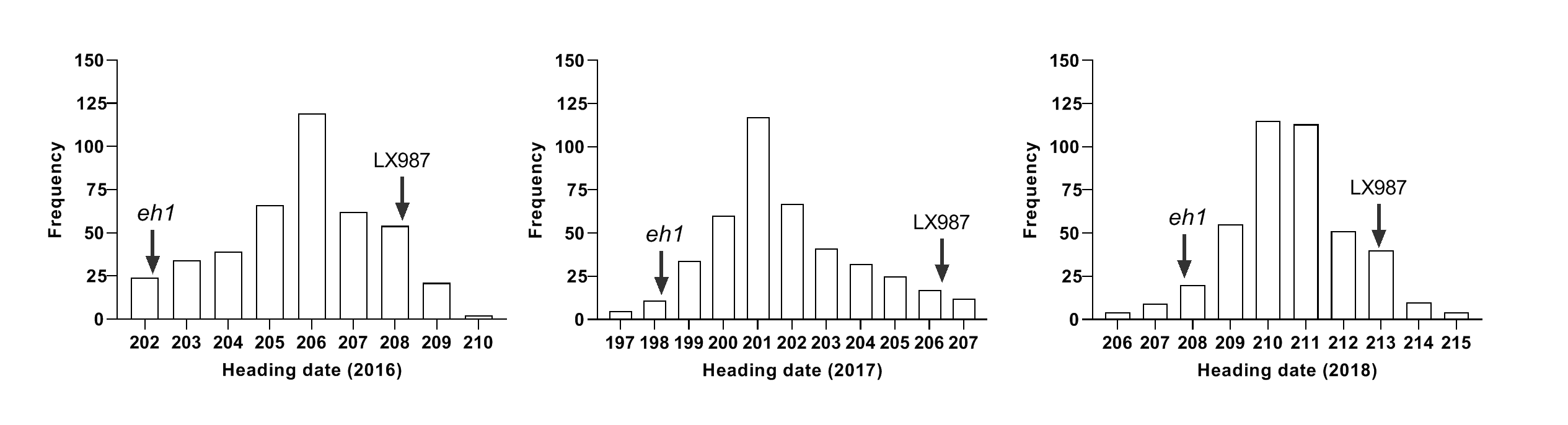

Supplement: Supplementary file 1 — Additional file 1: Fig S1. Distribution of heading date in the RIL population in 2016, 2017 and 2018. [file 12870_2020_2539_MOESM1_ESM.tif]

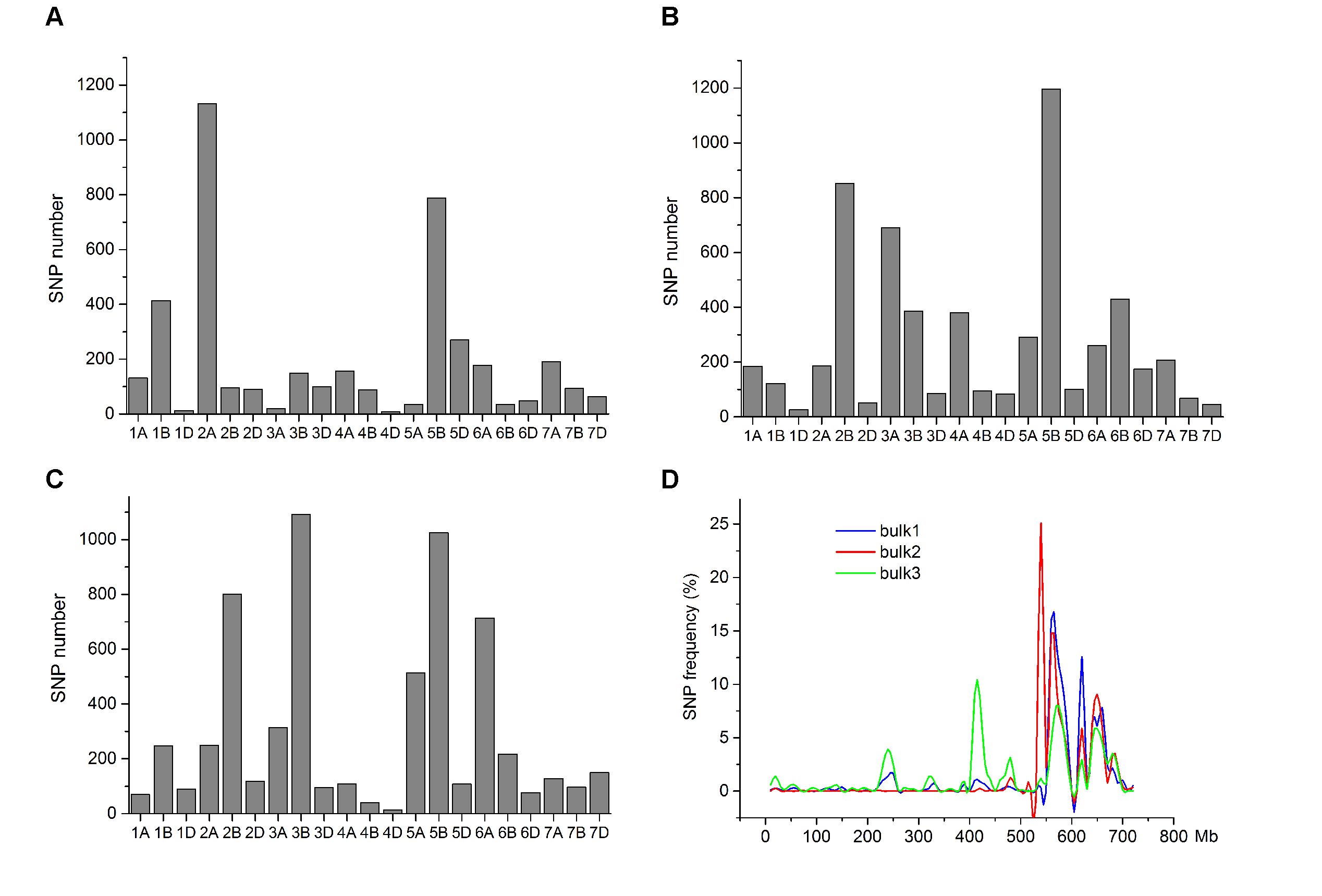

Supplement: Supplementary file 2 — Additional file 2: Fig S2. QTLs for HD in the RIL population detected by BSA analysis. (A-C) The number of SNP associated with HD on each chromosome between the early and late heading bulk 1 (A), bulk 2 (B), and bulk 3 (C). The QTL is present on chromosomes enriched higher number of selected SNP. (D) The frequency of SNP associated with HD distributed on chromosome 5B. The blue, red, and green lines represent bulk 1, bulk 2, and bulk 3, respectively. [file 12870_2020_2539_MOESM2_ESM.tif]

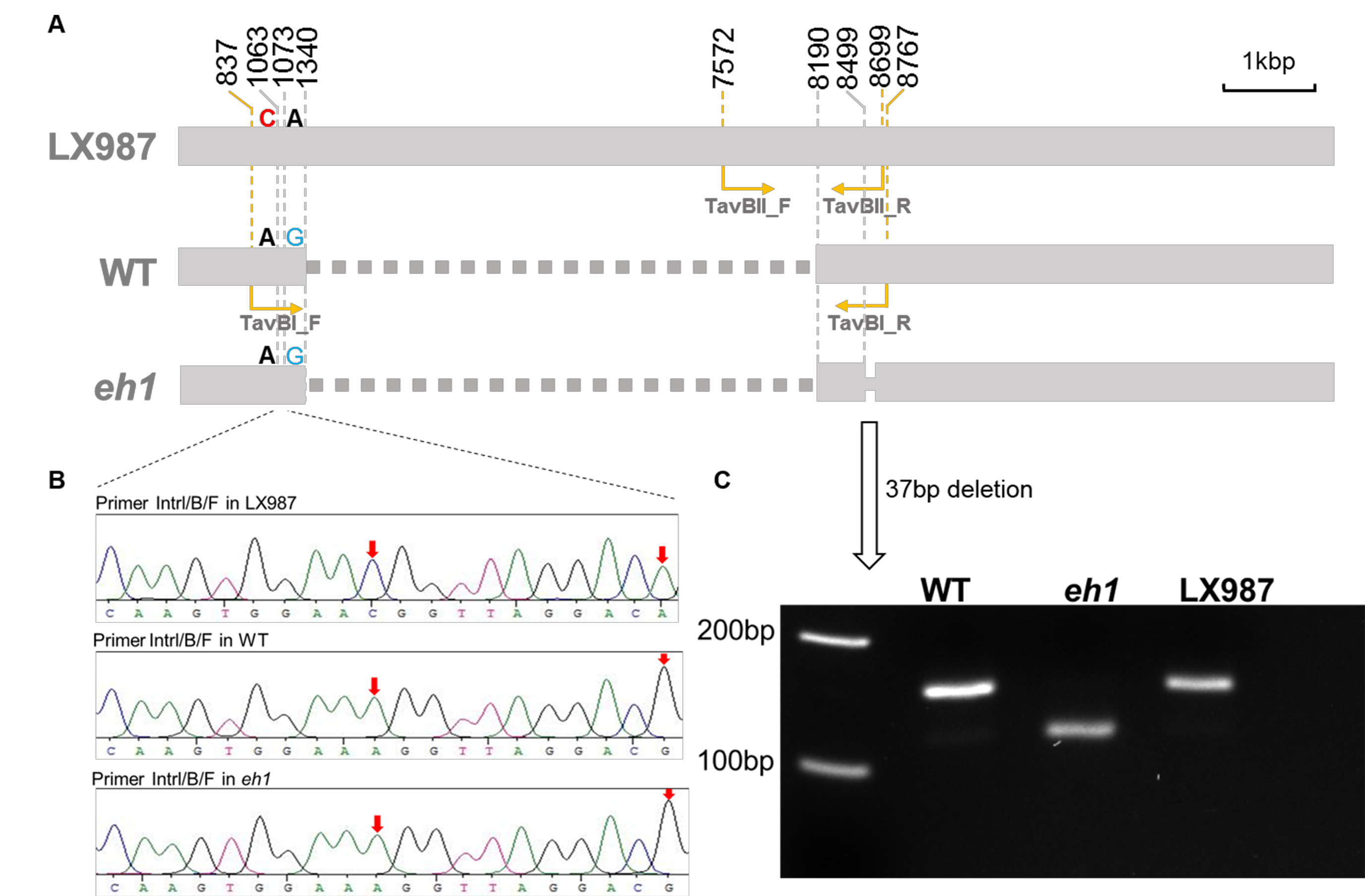

Supplement: Supplementary file 3 — Additional file 3: Fig S3. Sequence comparison of VRN-B1 in LX987, WT, and eh1. (A) Schematic representation of VRN-B1 in LX987, WT, and eh1. (B) Sequence analysis of primer Intrl/B/F in LX987, WT, and eh1. (C) Polymerase chain reaction amplification of a 37 bp deletion in eh1 by using specific primers. Full-length gels are presented in Supplementary Figure 10. [file 12870_2020_2539_MOESM3_ESM.tif]

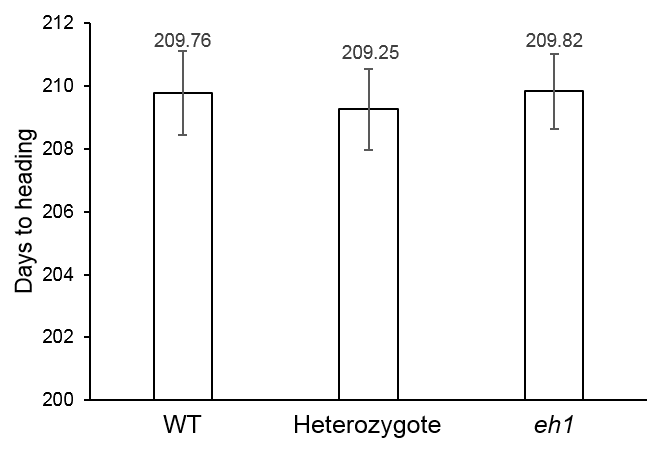

Supplement: Supplementary file 4 — Additional file 4: Fig S4. Days to heading of F2 individuals with or without 37 bp deletion of VRN-B1. WT indicates without 37 bp deletion while eh1 indicates with 37 bp deletion. [file 12870_2020_2539_MOESM4_ESM.tif]

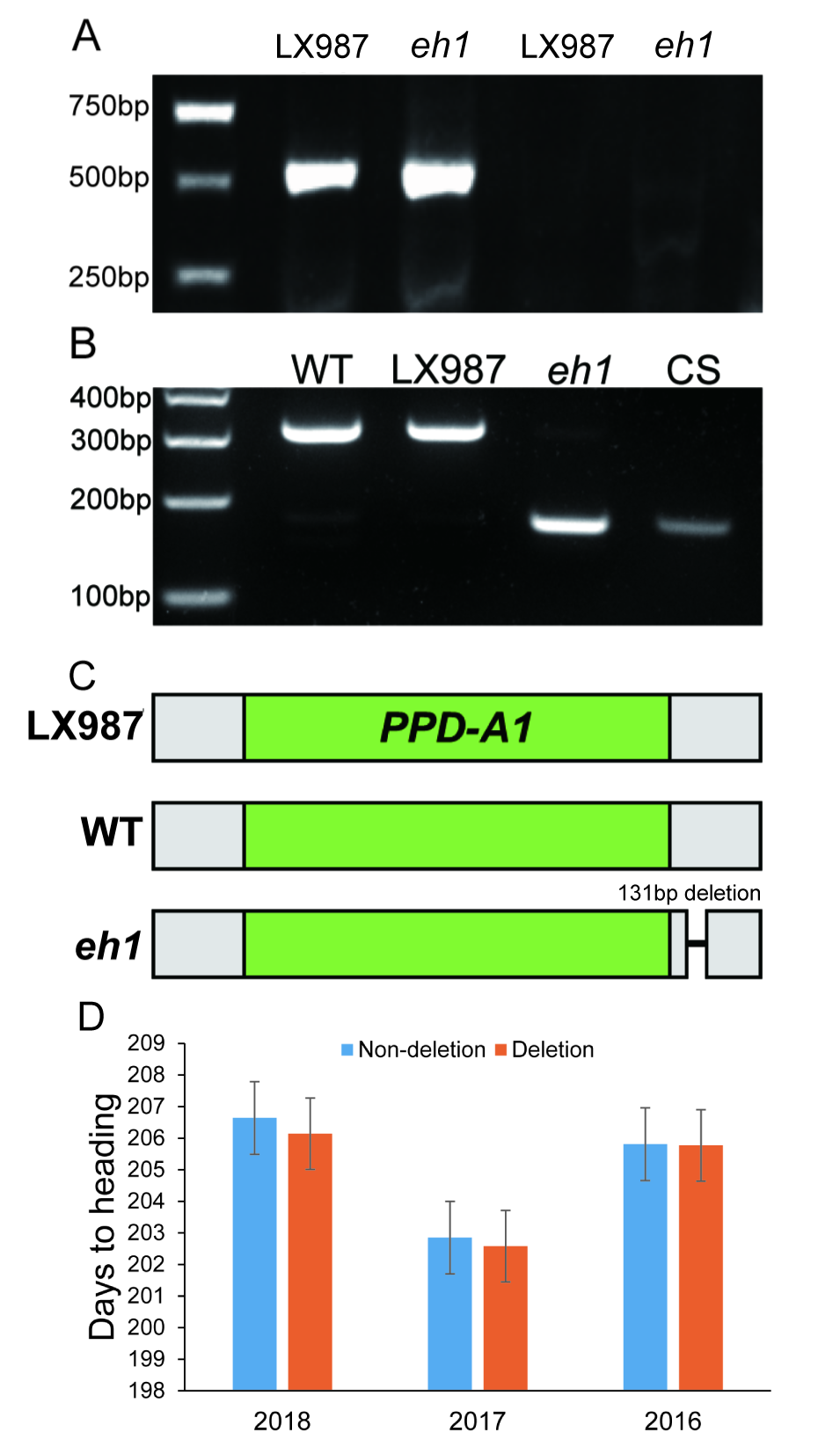

Supplement: Supplementary file 5 — Additional file 5: Fig S5. Variations of VRN-A1 and Ppd-A1 between LX987 and eh1. (A) Identification of natural variation of VRN-A1 in LX987 and eh1 by previously developed markers. Full-length gels are presented in Supplementary Figure 11. (B) Identification of the 131 bp deletion of Ppd-A1 in eh1 and Chinese Spring (CS). Full-length gels are presented in Supplementary Figure 12. (C) Sequence comparison of Ppd-A1 in LX987, WT, and eh1. (D) Days to heading of RILs with or without 131 bp deletion of Ppd-A1. [file 12870_2020_2539_MOESM5_ESM.tif]

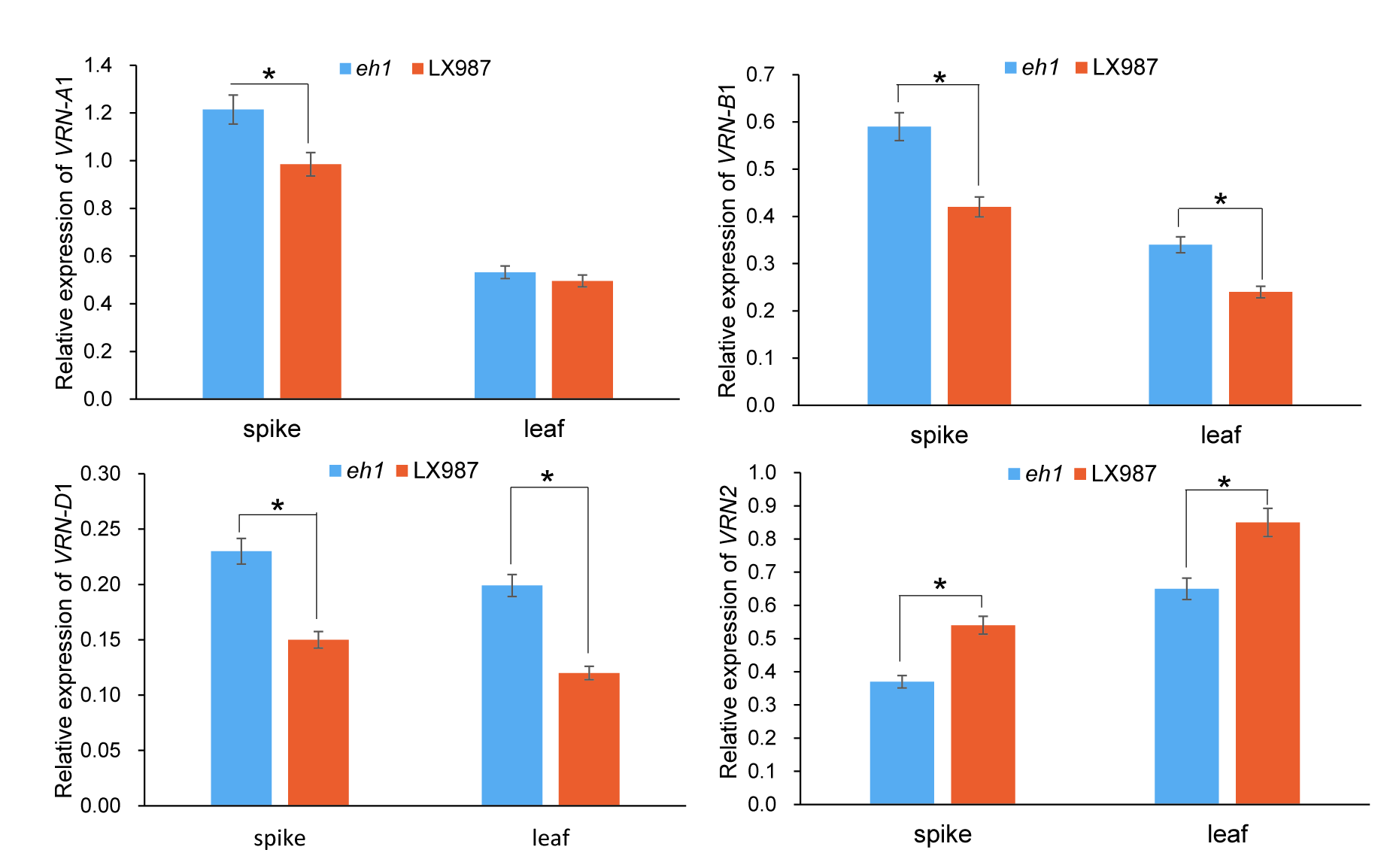

Supplement: Supplementary file 6 — Additional file 6: Fig S6. Relative expression levels of VRN-A1, VRN-B1, VRN-D1, and VRN2 genes in the young spikes and leaves of eh1 and LX987 when sampling at the same day. Student’s t-tests were used to assess the significance. *P < 0.05. [file 12870_2020_2539_MOESM6_ESM.tif]

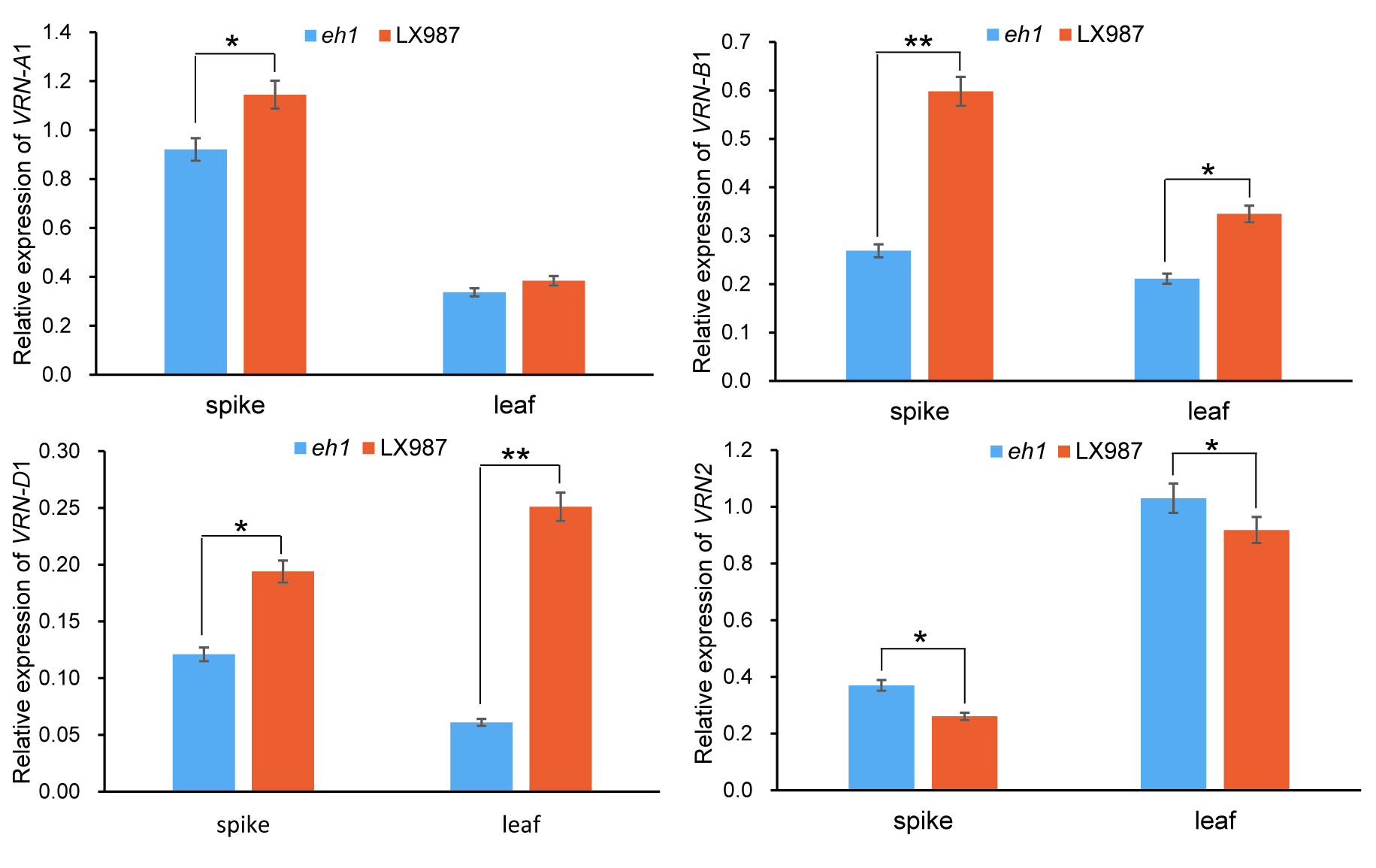

Supplement: Supplementary file 7 — Additional file 7: Fig S7. Relative expression levels of VRN-A1, VRN-B1, VRN-D1, and VRN2 genes in the young spikes and leaves of eh1 and LX987 when sampling at the same developmental stage. Student’s t-tests were used to assess the significance. **P < 0.01 and *P < 0.05. [file 12870_2020_2539_MOESM7_ESM.tif]

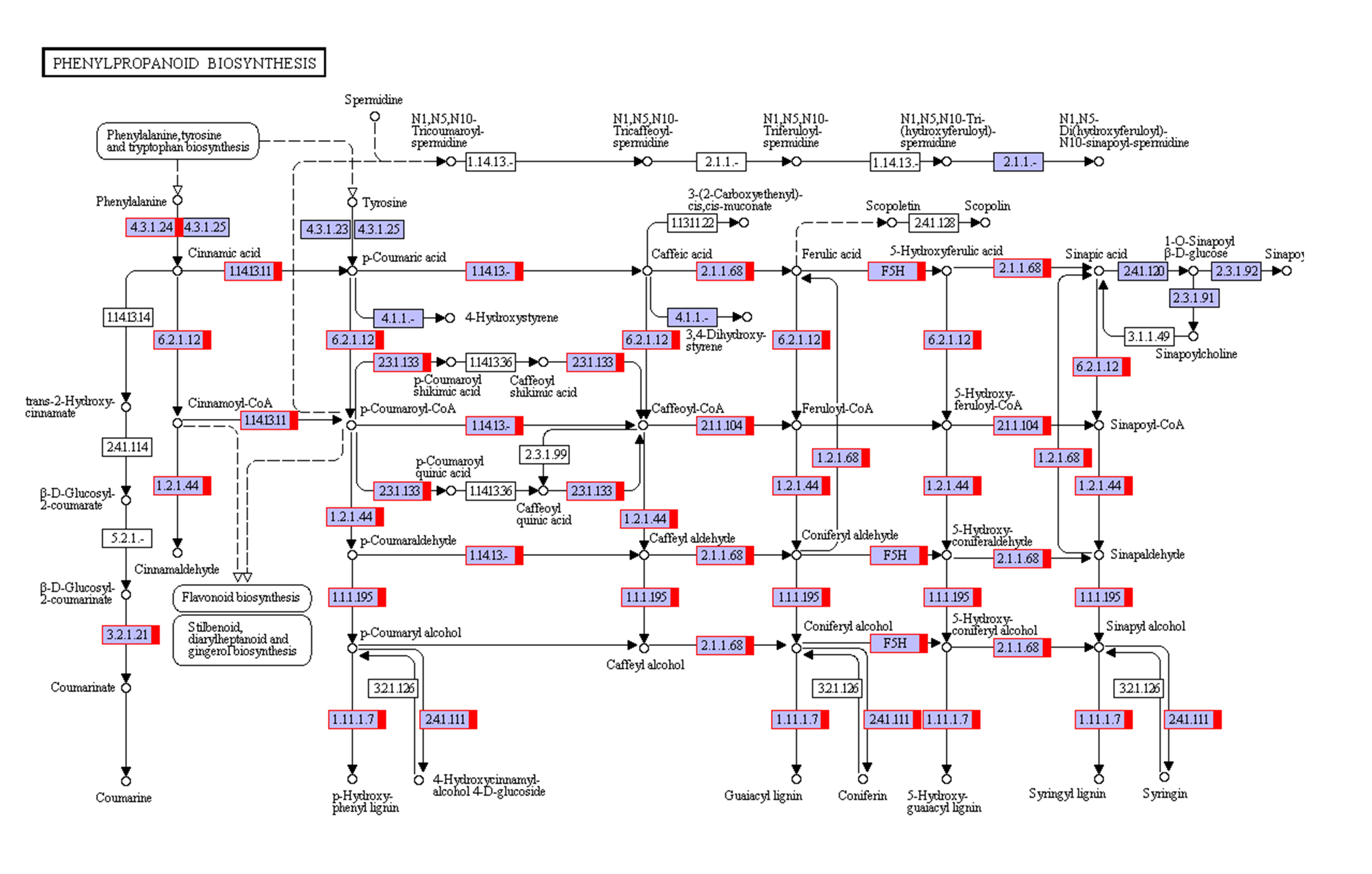

Supplement: Supplementary file 8 — Additional file 8: Fig S8. DEGs in phenylpropanoid biosynthesis. The red box filled with blue color indicates the up-regulated genes in the early heading bulk and the parent eh1, the black box filled with blue color represents down-regulated genes, and the box without color is genes showing no expression difference. [file 12870_2020_2539_MOESM8_ESM.tif]

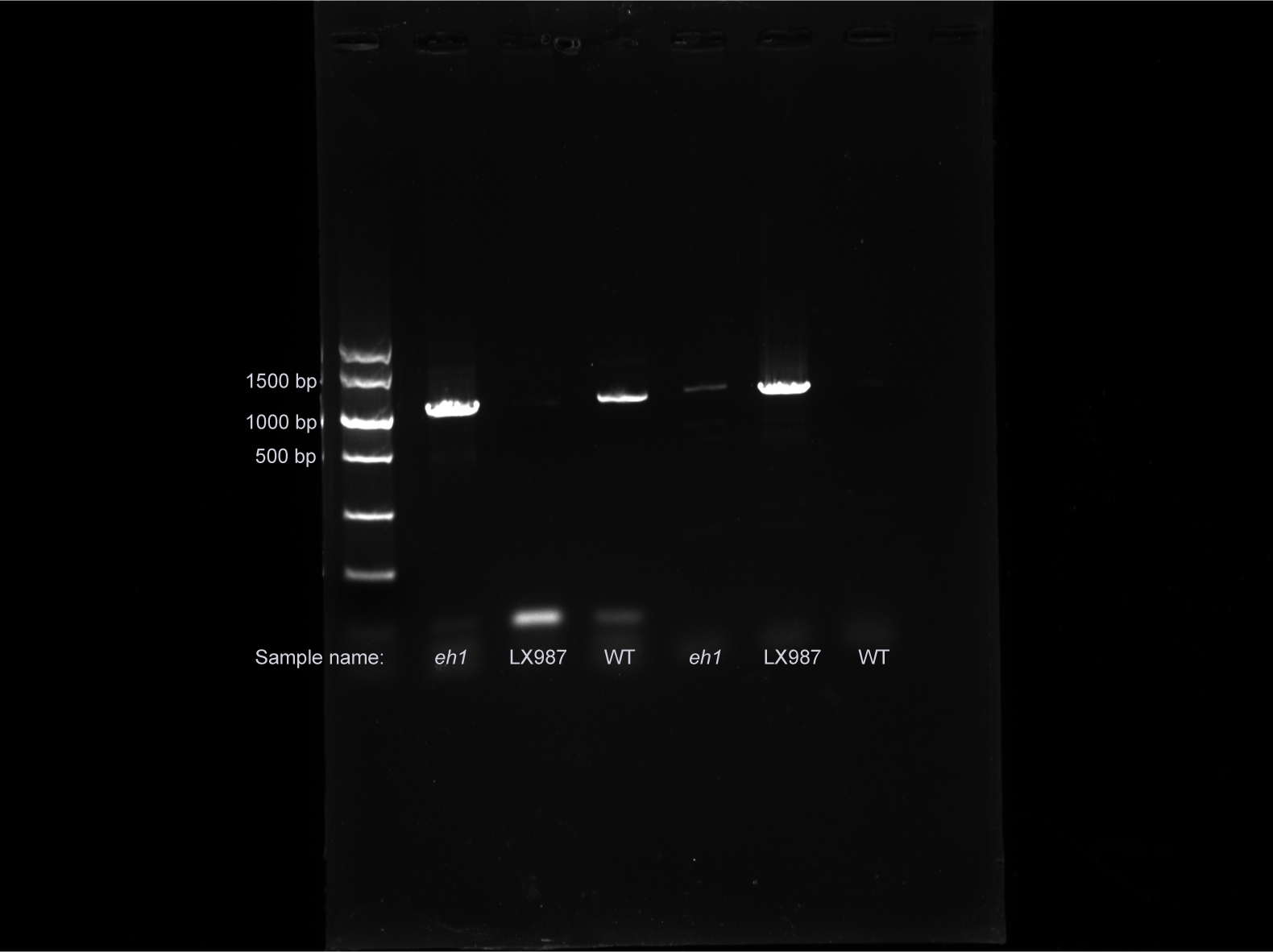

Supplement: Supplementary file 9 — Additional file 9: Fig S9. Original gel image of Fig. 4a. [file 12870_2020_2539_MOESM9_ESM.tif]

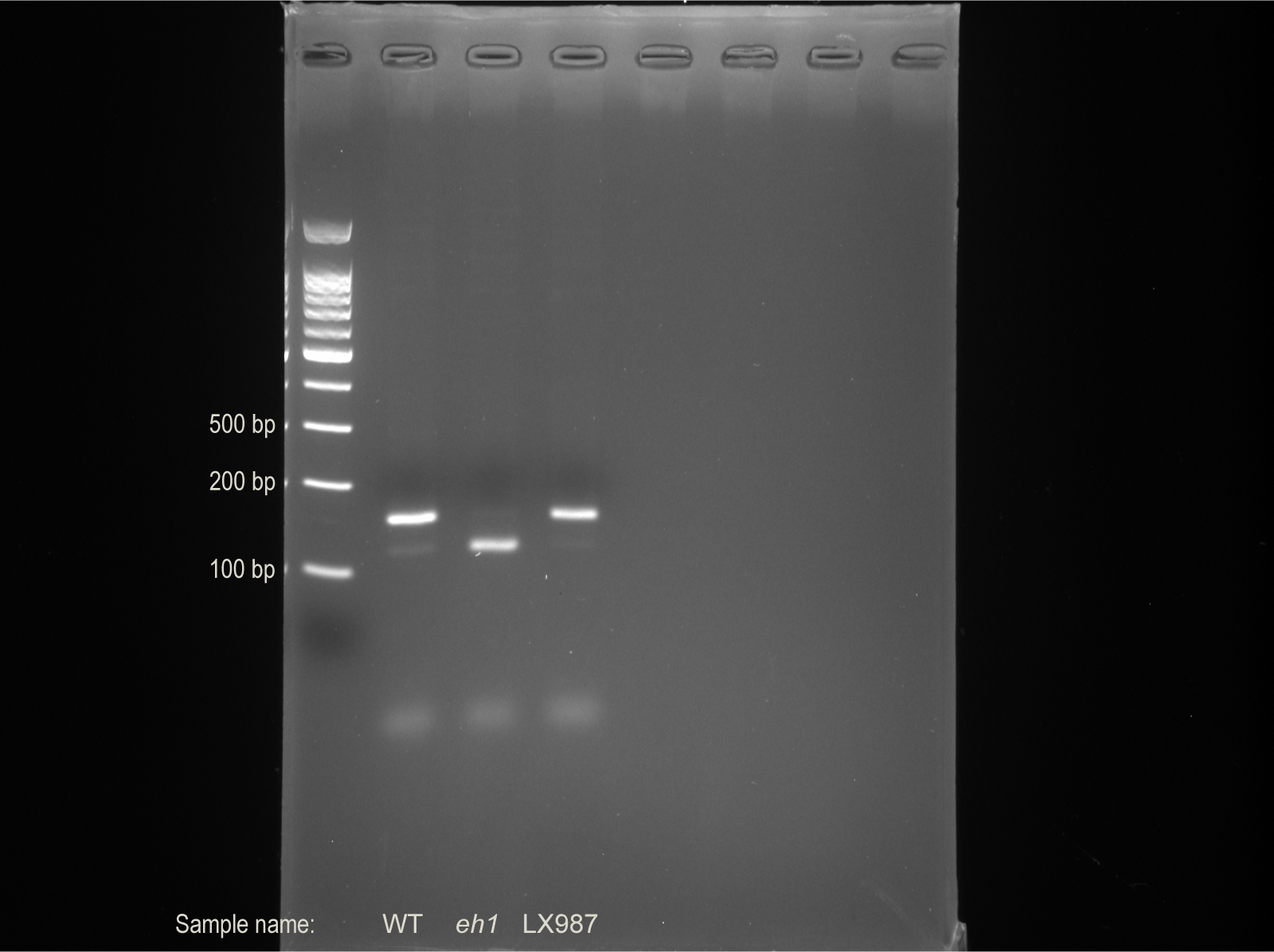

Supplement: Supplementary file 10 — Additional file 10: Fig S10. Original gel image of Fig S3C. [file 12870_2020_2539_MOESM10_ESM.tif]

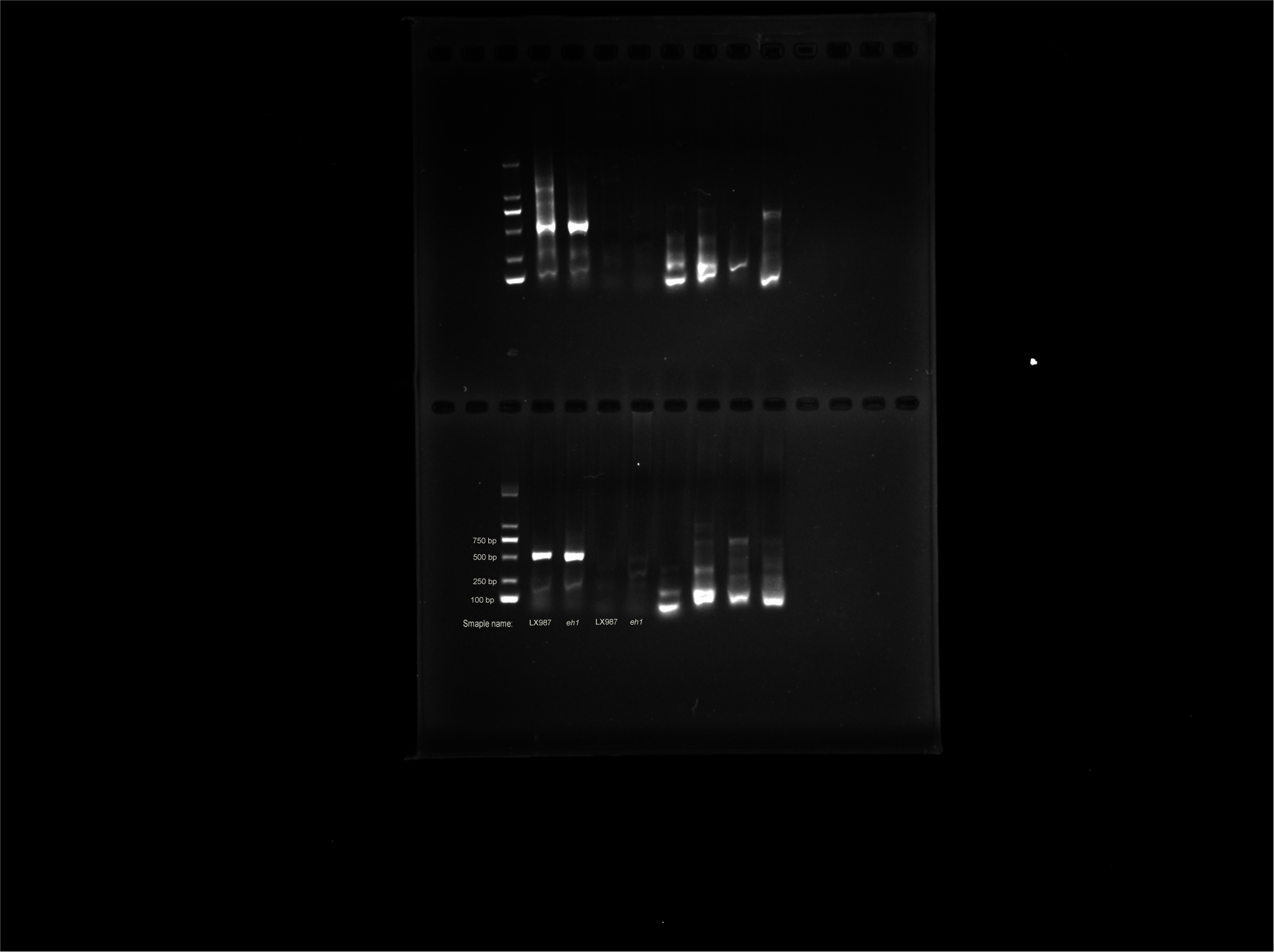

Supplement: Supplementary file 11 — Additional file 11: Fig S11. Original gel image of Fig S5A. [file 12870_2020_2539_MOESM11_ESM.tif]

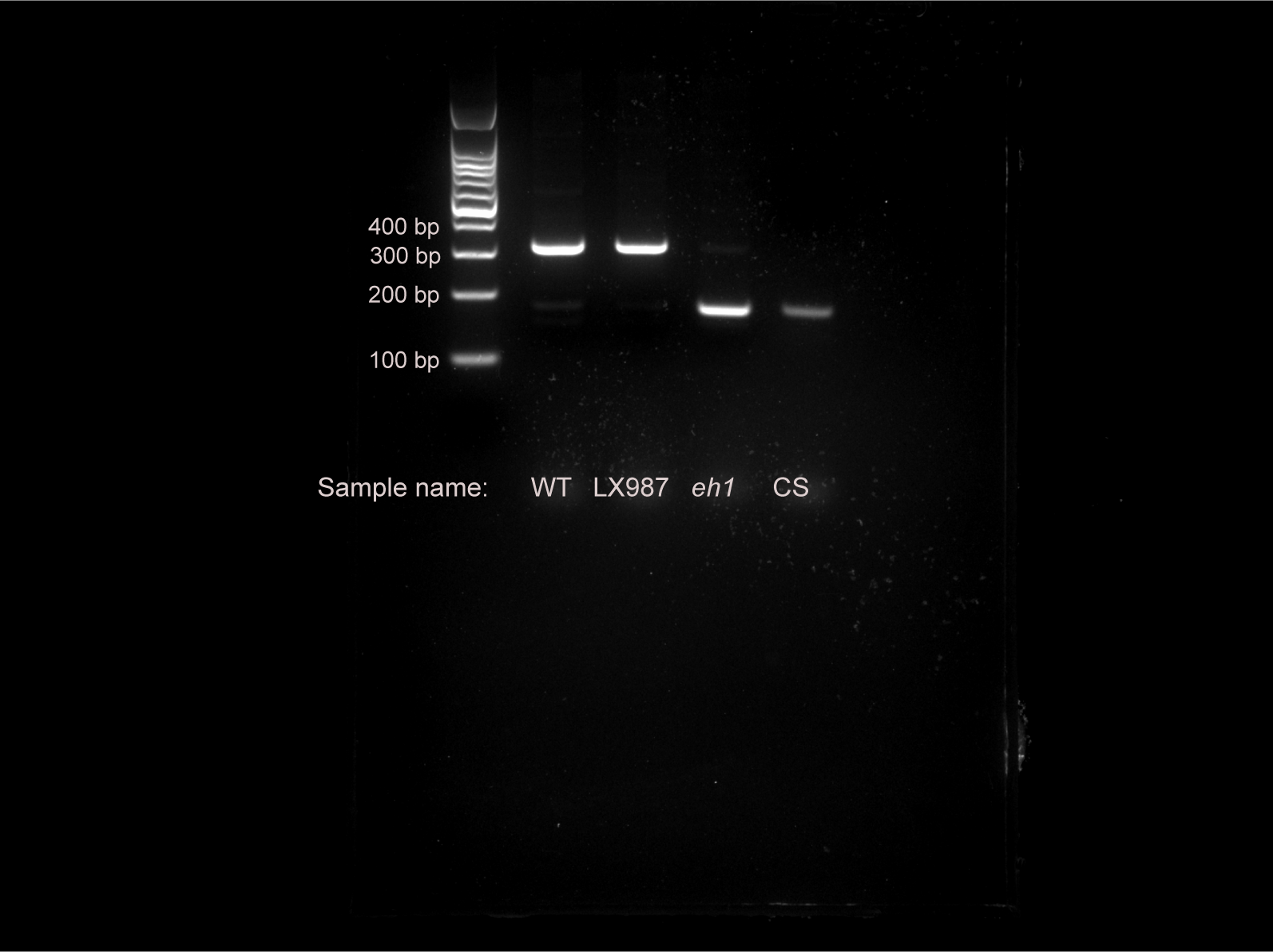

Supplement: Supplementary file 12 — Additional file 12: Fig S12. Original gel image of Fig S5B. [file 12870_2020_2539_MOESM12_ESM.tif]
